# Loss of *Grin2a* causes a transient delay in the electrophysiological maturation of hippocampal parvalbumin interneurons

**DOI:** 10.1038/s42003-023-05298-9

**Published:** 2023-09-19

**Authors:** Chad R. Camp, Anna Vlachos, Chiara Klöckner, Ilona Krey, Tue G. Banke, Nima Shariatzadeh, Sarah M. Ruggiero, Peter Galer, Kristen L. Park, Adam Caccavano, Sarah Kimmel, Xiaoqing Yuan, Hongjie Yuan, Ingo Helbig, Tim A. Benke, Johannes R. Lemke, Kenneth A. Pelkey, Chris J. McBain, Stephen F. Traynelis

**Affiliations:** 1 Department of Pharmacology and Chemical Biology, Emory University School of Medicine, Atlanta, GA 30322 USA; 2 Section on Cellular and Synaptic Physiology, Eunice Kennedy-Shriver National Institute of Child Health and Human Development, National Institutes of Health, Bethesda, MD 20892 USA; 3 Institute of Human Genetics, University of Leipzig Medical Center, Leipzig, Germany; 4 Division of Neurology, Children’s Hospital of Philadelphia, Philadelphia, PA 19104 USA; 5 The Epilepsy NeuroGenetics Initiative, Children’s Hospital of Philadelphia, Philadelphia, PA 19104 USA; 6 Department of Biomedical and Health Informatics, Children’s Hospital of Philadelphia, Philadelphia, PA 19146 USA; 7 University of Colorado School of Medicine and Children’s Hospital Colorado, Aurora, CO 80045 USA; 8 Center for Functional Evaluation of Rare Variants, Emory University School of Medicine, Atlanta, GA 30322 USA; 9 Department of Neurology, University of Pennsylvania Perelman School of Medicine, Philadelphia, PA 19104 USA; 10 Center for Rare Diseases, University of Leipzig Medical Center, Leipzig, Germany; 11 Center for Neurodegenerative Disease, Emory University School of Medicine, Atlanta, GA 30322 USA

**Keywords:** Ion channels in the nervous system, Neuronal development, Epilepsy

## Abstract

N-methyl-D-aspartate receptors (NMDARs) are ligand-gated ionotropic glutamate receptors that mediate a calcium-permeable component to fast excitatory neurotransmission. NMDARs are heterotetrameric assemblies of two obligate GluN1 subunits (*GRIN1*) and two GluN2 subunits (*GRIN2A*-*GRIN2D*). Sequencing data shows that 43% (297/679) of all currently known NMDAR disease-associated genetic variants are within the *GRIN2A* gene, which encodes the GluN2A subunit. Here, we show that unlike missense *GRIN2A* variants, individuals affected with disease-associated null *GRIN2A* variants demonstrate a transient period of seizure susceptibility that begins during infancy and diminishes near adolescence. We show increased circuit excitability and CA1 pyramidal cell output in juvenile mice of both *Grin2a*^*+/−*^ and *Grin2a*^*−/−*^ mice. These alterations in somatic spiking are not due to global upregulation of most *Grin* genes (including *Grin2b*). Deeper evaluation of the developing CA1 circuit led us to uncover age- and *Grin2a* gene dosing-dependent transient delays in the electrophysiological maturation programs of parvalbumin (PV) interneurons. We report that *Grin2a*^*+/+*^ mice reach PV cell electrophysiological maturation between the neonatal and juvenile neurodevelopmental timepoints, with *Grin2a*^*+/−*^ mice not reaching PV cell electrophysiological maturation until preadolescence, and *Grin2a*^*−/−*^ mice not reaching PV cell electrophysiological maturation until adulthood. Overall, these data may represent a molecular mechanism describing the transient nature of seizure susceptibility in disease-associated null *GRIN2A* patients.

## Abstract (web summary)

Null *GRIN2A* human patients display a largely transient seizure burden that resolves with age, which may be attributable to a transient delay in the developmental maturation of parvalbumin-positive interneurons in CA1 as is observed in *Grin2a*^+/−^ and *Grin2a*^*-*/-^ mice.

## Introduction

N-methyl-D-aspartate receptors (NMDARs) comprise a family of ligand-gated ionotropic glutamate receptors that mediate a calcium-permeable component to fast excitatory neurotransmission^1^. NMDARs are heterotetrameric assemblies of two obligate GluN1 subunits (encoded by the *GRIN1* gene) and two GluN2 subunits (encoded by the *GRIN2A*-*GRIN2D* genes)^1^. Given their ubiquitous expression, participation in glutamatergic neurotransmission, and facilitation of calcium entry into cells, NMDARs have been implicated in a host of physiological and developmental roles including learning, memory, spatial navigation, coordinated movement, decision making, neuronal migration, morphological development, and synaptic connectivity^1–9^.

A growing volume of sequencing data implicates genetic variation within NMDARs as a contributing factor to neuropathological conditions including epilepsy, schizophrenia, autism, intellectual disability, and developmental delay^10,11^. These genetic variants, which are absent from the healthy population, illustrate a critical role for NMDARs in basic and higher-level cognitive function^12,13^. Moreover, when stratified by subunit, 43% (297/679) of all currently known NMDAR disease-associated genetic variants are within the *GRIN2A* gene, which encodes the GluN2A subunit^1,10^. Neurological conditions associated with heterozygous *GRIN2A* variation present with a range of symptoms, with the most common being epilepsy and intellectual disability, coupled with some form of speech disorder, most notably oral motor apraxia^1,10,14^. In addition, heterozygous disease-associated variants in the *GRIN2A* gene have been identified as a high-risk factor for schizophrenia in genome-wide association studies^15,16^. Nearly one-third (98/297) of heterozygous disease-associated *GRIN2A* variants represent null variants^1^, mainly mediated by nonsense variants and deletions, in which no functional GluN2A protein would be made by the affected allele. Here, we show that unlike those with loss-of-function or gain-of-function missense *GRIN2A* variants, the majority of individuals affected with disease-associated null *GRIN2A* variants demonstrate a transient period of seizure susceptibility that begins during infancy and diminishes near adolescence.

To investigate the cellular mechanisms for this transient seizure susceptibility, we used global *Grin2a*^+/−^ and *Grin2a*^−/−^ mice as models for null *GRIN2A* variants. While individuals with null *GRIN2A* variants are usually haploinsufficient (but see ref. ^17^), current data on *Grin2a*^+/−^ mice is limited. *Grin2a*^−/−^ mice, however, display neurological characteristics similar to individuals affected with null *GRIN2A* variants, such as transient cortical epileptiform discharges and deficits in spatial learning^18,19^. Given that this transient seizure susceptibility manifests early in life, we hypothesized that this may be a neurodevelopmental disease, in which aberrant circuit activity during the critical plasticity period of development disrupts the excitatory-to-inhibitory balance. Loss of early GluN2A signaling would promote profound network disruptions as the GluN2B-to-GluN2A switch, a period in which the relative ratio of GluN2B:GluN2A transcript tilts in favor of GluN2A being in the majority, confers cells with faster NMDAR-mediated excitatory postsynaptic currents and less overall calcium transfer per synaptic event^20–25^. This change in postsynaptic calcium signaling coincides with dynamic periods of neurodevelopment in which cells display morphological changes, synaptic connections are established and pruned, and various ion channels regulating cellular excitability are upregulated^26–28^.

The GluN2A subunit is expressed in excitatory glutamatergic pyramidal cells^1,29^ and multiple interneuron subtypes^29^. Inhibition or targeted knockdown of the GluN1, GluN2B, or GluN2D subunits impede interneuron development, suggesting an active role for NMDARs in interneuron function and maturation^30–33^. Given the transient nature of seizure susceptibility observed in null *GRIN2A* patients, the temporal expression pattern of GluN2A, and potential roles of NMDARs in circuit refinement and interneuron maturation, we hypothesized that the reduced GluN2A signaling may impact interneuron function and thereby contribute to the formation of a transiently hyperexcitable network. Our data suggest that *Grin2a*^*+/−*^ and *Grin2a*^*−/−*^ mice show a delay in parvalbumin-positive (PV) interneuron maturation, with resolution of aberrant interneuron function occurring at a time—post adolescence—roughly corresponding to the time null *GRIN2A* variant patients show seizure offset. These data suggest a molecular mechanism for the transient seizure susceptibility observed in null *GRIN2A* patients and provide further evidence for GluN2A’s role in circuit maturation.

## Methods

### Animals and breeding

All procedures involving the use of animals performed at Emory University were reviewed and approved by the Emory University IACUC, and were performed in full accordance with state and federal Animal Welfare Acts and Public Health Service policies. *Grin2a*^−/−^ mice were obtained from the laboratory of Masayoshi Mishina (University of Tokyo, Japan) and were generated via insertion of a neomycin resistance gene and Pau sequence (mRNA destabilizing and transcription-pausing signals) into the coding region of the transmembrane domain of the *Grin2a* gene^19^. These mice were then backcrossed more than 15 times to a C57BL/6J (Jax stock number: 00664) background at Emory before use. Mice were genotyped by performing PCRs for the neomycin cassette (forward: GGGCGCCCGGTTCTT; reverse: CCTCGTCCTGCAGTTCATTCA) and the WT *Grin2a* gene (forward: GCCCGTCCAGAATCCTAAAGG; reverse: GCAAAGAAGGCCCACACTGATA). Heterozygous *Grin2a*^+/−^ mice were identified as being positive for both probes.

In order to visualize PV cells for use in electrophysiological experiments, *Grin2a*^−/−^ mice were crossed with *Pvalb*-tdTomato mice (Jax stock number: 027395 – Tg(*Pvalb*-tdTomato15Gfng)) to generate *Grin2a*^*+/+*^:*Pvalb*-tdTom, *Grin2a*^*+/−*^:*Pvalb*-tdTom, and *Grin2a*^*−/−*^:*Pvalb*-tdTom mice. This reporter line has already been backcrossed to a C57BL/6J background and has been previously validated to be selective and specific for PV^+^-GABAergic interneurons, including those within the CA1 subfield^34,35^. For identification of neonatal PV cells in acutely prepared hippocampal tissue, *Tac1*-Cre (Jax stock number: 021877 – B6;129S-*Tac1*^tm1.1(cre)Hze^/J) driver mice were crossed with eGFP-Floxed (Jax stock number: 004077 – B6;129-Gt(ROSA)26Sor^tm2Sho^/J) mice to produce *Tac1*-Cre:eGFP mice. *Tac1* has previously been described to be specifically expressed in PV cells, with no expression in MGE-derived somatostatin-positive cells^36,37^. In addition, since CA1 *stratum oriens* and *stratum pyramidale* had the most extensive overlap for PV and Tac1 (see Supplemental Fig. S5), all recordings made from *Tac1*-positive cells were chosen in these two layers only.

The following definitions are used for mice of various ages: neonatal (P6-8), juvenile (P13-15), preadolescent (P20-26), and adult (P70-125)^38^. All mice were maintained in a conventional vivarium, given standard chow and water ad libitum, with a 12-h light cycle. Both male and female mice were used in all experiments.

### Human patient data

Deidentified data on seizure onset and offset were obtained from consented patients under protocols approved by the University of Colorado IRB (COMIRB 16-1520), the Children’s Hospital of Philadelphia IRB, or University of Leipzig IRB (224/16-ek and 379/21-ek). Seizure offset was defined as the age of last seizure. All cases are currently ongoing and all relevant ethical regulations regarding the gathering and reporting of human patient deidentified data have been followed.

### Gene expression analysis

After juvenile (P13-15) *Grin2a*^*+/+*^, *Grin2a*^*+/−*^, and *Grin2a*^*−/−*^ mice were overdosed with inhaled isoflurane, whole hippocampi from both hemispheres were removed and placed into an Eppendorf tube and flash frozen in liquid nitrogen. A total of 18 samples were collected, with six replicates per genotype. After tissue collection, RNA was extracted using the miRNeasy Mini Kit (Qiagen; 217004) according to kit instructions. RNA quality was assessed via an Agilent 2100 Bioanalyzer and reported as RNA Integrity Numbers (RINs) in Supplemental Table S3. Next, multiplex mRNA expression analysis was performed using a custom designed NanoString nCounter panel consisting of all seven *GRIN* genes. Data were analyzed using NanoString’s nSolver module where samples were checked for internal quality control (QC) metrics such as imaging QC, binding density QC, positive control linearity, and positive control limit of detection (see Supplemental Table S3 for details). Samples were background subtracted using negative controls (eight different hybridization probes for which no transcript has been supplied) and data that did not meet the minimum detectable threshold of 30 counts were excluded. Counts for the *Grin3b* gene were excluded from analysis for failing to meet the limit of detection. Samples were normalized to six positive hybridization controls (at the following concentrations in the 30 μL hybridization reaction: 128 fM, 32 fM, 8 fM, 2 fM, 0.5 fM, and 0.125 fM) and 11 housekeeping genes (*Aars*, *Asb10*, *Ccdc127*, *Cnot10*, *Csnk2a2*, *Fam104a*, *Gusb*, *Lars*, *Mto1*, *Supt7l*, *Tada2b*). Normalized datasets were compared for significance using the Benjamini-Yekutieli method for controlling false discovery rate.

### Acute hippocampal slice preparation and electrophysiological recordings

After mice were overdosed with inhaled isoflurane, brains were rapidly removed and immediately placed in ice-cold sucrose-based artificial cerebrospinal fluid (aCSF) containing the following (in mM): 88 sucrose, 80 NaCl, 2.5 KCl, 1.25 Na_2_HPO_4_, 26 NaHCO_3_, 10 glucose, 2 thiourea, 3 sodium pyruvate, 5 sodium ascorbate, 12 N-acetylcysteine, 10 MgSO_4_, and 0.5 CaCl_2_ bubbled in 95% O_2_/5% CO_2_. 300-µm thick horizontal, ventral hippocampal slices were made using a vibratome (Lecia, VT-1200S) and slices were incubated in a sucrose-based aCSF as described above but with 4 mM MgSO_4_ at 32 °C for 30 min then returned to room temperature for at least an hour before use. All recordings were made in the following aCSF extracellular solution (in mM): 126 NaCl, 2.5 KCl, 1.25 Na_2_HPO_4_, 26 NaHCO_3_, 20 glucose, 1.5 MgSO_4_, and 1.5 CaCl_2_ bubbled with 95% O_2_/5% CO_2_ and held at 30–32 °C using an inline heater (Warner, SH-27B). Cells were visualized using an upright Olympus BX50W microscope with IR-DIC optics coupled to a Dage IR-2000 camera. Whole-cell patch clamp recordings were obtained using an Axopatch 200B (Molecular Devices) or a Multiclamp 700B (Molecular Devices), digitized at 20 kHz using a Digidata 1440a (Molecular Devices) controlled by pClamp 10.6 software (Molecular Devices). All signals were low-pass filtered at 2 kHz using a Bessel 8-pole filter (Warner, LPF-8). Patch clamp electrodes were pulled using a Sutter P1000 horizontal puller from thin-walled borosilicate capillary tubes (WPI), with a typical resistance of 4–8 MΩ.

For current clamp recordings (action potential spiking probability, intrinsic excitability, and action potential firing properties), the following intracellular solution was used (in mM): 115 potassium gluconate, 0.6 EGTA, 2 MgCl_2_, 2 Na_2_ATP, 0.3 Na_2_GTP, 10 HEPES, 5 sodium phosphocreatine, 8 KCl, and 0.3–0.5% biocytin. After obtaining a whole-cell configuration, all cells were allowed to dialyze for 5 min in current-clamp mode. The liquid junction potential was not corrected and all current-clamp responses were automatically bridge-balanced using the Multiclamp 700B clamp commander software. For action potential spiking probability experiments, CA1 pyramidal cells were held at −60 mV by injecting ±10–30 pA of current. Any CA1 pyramidal cells that fired spontaneous action potentials at −60 mV were rejected from analysis. A monopolar iridium-platinum stimulating electrode (FHC, Inc.) was placed in the upper 1/3rd of the Schaffer collaterals to elicit a single 50 µs stimulation at a frequency of 0.03 Hz. This stimulation paradigm was used to find a stimulation intensity that would be just below a threshold that would produce a single action potential in the patched CA1 pyramidal cell held at −60 mV in current-clamp mode, with a typical stimulation intensity of 40–70 µA. Once this stimulation intensity was established, the stimulation paradigm was shifted to deliver five 50 µs bursts at a frequency of 100 Hz every 30 s^39^. A total of five epochs were recorded per cell with action potential spiking probability calculated per stimulation number as number of spikes/5. Input resistance was calculated using the slope of voltage deflections in response to 500 ms current injections of −200, −150, −100, −50, 0, and 50 pA, with an inter-sweep interval of 2 s. The membrane time constant was calculated in response 20–30 sweeps of a 500 ms, −50 pA current injection, with an inter-sweep interval of 2 s. Composite responses were made by averaging 20–25 traces together using Clampfit (Molecular Devices). A weighted time constant was calculated using formula (1) by fitting a dual-exponential to each response in ChanneLab (Synaptosoft). Rheobase was calculated in response to 500 ms current injections starting at 0 pA and increasing by 2 pA every 2 s. Rheobase was defined as the minimal current injection required to elicit an action potential during the current injection period. Action potential firing frequency was calculated in response a 500 ms current injection every 2 s starting at −100 pA, and increasing by 20 pA for neonate and juvenile mice and 50 pA for preadolescent and adult mice, until the cell displayed depolarization-induced block of firing. Number of action potentials per current injection were calculated using pClamp (Molecular Devices). Action potential half-width, action potential amplitude, action potential threshold, and afterhyperpolarization amplitude were calculated using pClamp (Molecular Devices) from action potentials obtained during rheobase recordings.

For voltage clamp experiments (NMDAR-mediated EPSCs), the following intracellular solution was used (in mM): 100 Cs-gluconate, 5 CsCl, 0.6 EGTA, 5 BAPTA, 5 MgCl_2_, 8 NaCl, 2 Na-ATP, 0.3 Na-GTP, 40 HEPES, 5 Na-phosphocreatine, and 3 QX-314. A monopolar iridium-platinum stimulating electrode (FHC, Inc.) was placed in the upper 1/3rd of the Schaffer collaterals to elicit a single 50 µs stimulation at a frequency of 0.03 Hz and the NMDAR-mediated EPSC was pharmacologically isolated with 10 μM NBQX and 10 μM gabazine. Cells were held at +40 mV and stimulation intensity was chosen to be near 50% of the maximum peak amplitude of the EPSC. A total of 8–12 epochs were recorded and averaged together. At the conclusion of recording, 200 μM DL-APV was applied to ensure responses were mediated via NMDARs. A weighted time constant was calculated using the following formula by fitting a dual-exponential function to each composite mIPSC trace in ChanneLab (Synaptosoft):

1 $${{{\tau }}}_{w}=	 {{{\tau }}}_{{{{{{\rm{FAST}}}}}}}\left[{{{{{{{\rm{amplitude}}}}}}}}_{{{{{{{\rm{FAST}}}}}}}}/\left({{{{{{{\rm{amplitude}}}}}}}}_{{{{{{{\rm{FAST}}}}}}}}+{{{{{{{\rm{amplitude}}}}}}}}_{{{{{{{\rm{SLOW}}}}}}}}\right)\right] \\ 	 +{{{\tau }}}_{{{{{{{{\rm{SLOW}}}}}}}}}\left[{{{{{{{\rm{amplitude}}}}}}}}_{{{{{{{\rm{SLOW}}}}}}}}/\left({{{{{{{\rm{amplitude}}}}}}}}_{{{{{{{\rm{FAST}}}}}}}}+{{{{{{{\rm{amplitude}}}}}}}}_{{{{{{{\rm{SLOW}}}}}}}}\right)\right]$$

where *τ*_FAST_ is the fast deactivation time constant, *τ*_SLOW_ is the slow deactivation time constant, amplitude_FAST_ is the current amplitude of the fast deactivation component, and amplitude_SLOW_ is the current amplitude of the slow deactivation component.

For all electrophysiological recordings, series resistance was monitored throughout all experiments and was typically 8−20 MΩ. For current clamp recordings, cells were briefly switched to voltage clamp and held at −60 mV while a series of 50 ms, 5 mV square waveforms were applied to the cell. For voltage clamp recordings, this 50 ms, 5 mV square wave was included in the stimulation protocol. Series resistance was monitored throughout the entire recording, while current clamp recordings had series resistance measurements made at the beginning and at the end of each experiment, which usually lasted 5–7 min total. All series resistances were measured offline by analyzing the peak of the capacitive charging spike and applying Ohm’s law. If the series resistance changed >25% during the experiment, or ever exceeded 30 MΩ, then the cell was excluded.

### Interneuron anatomical reconstructions

After biocytin filling during whole-cell recordings, slices were fixed with 4% PFA in PBS overnight, then permeabilized with 0.3% Triton-X in PBS and incubated with streptavidin Alexa546 (Invitrogen, S11225; 1:500). Resectioned slices (75 µm) were mounted on gelatin-coated slides using Mowiol mounting medium. We also found similar axonal recovery success by filling cells with 0.5% biocytin and permeabilizing slices with 1.2% Triton-X in PBS for 10 min prior to incubation in streptavidin AlexaFluor546 without resectioning. Cells were visualized using epifluorescence microscopy and images for representative examples were obtained with confocal microscopy. Cells were reconstructed and analyzed with Sholl analysis using Neurolucida software (MBF Bioscience). Polar histograms of dendrites and axons were created using the Neurolucida function (10 degree bins). Polarity preference was determined by calculating the percentage of horizontally (150–210, 330–30 degrees) or vertically (60–120, 240–300 degrees) oriented axons for each genotype.

### Immunohistochemistry for GABAergic interneuron markers

After mice were overdosed with inhaled isoflurane, they were transcardially perfused with cold phosphate-buffered saline (PBS; pH 7.35), and subsequently perfused with cold 4% paraformaldehyde (PFA) in PBS. Brains were removed and fixed for 24 h in 4% PFA in PBS before being transferred to a 30% sucrose solution dissolved in PBS until the brains sank. After cryoprotection, brains were frozen in optimal cutting temperature solution (OCT, Fisher) and serial 50 µm coronal hippocampal sections were obtained, with a total of five sections per animal, spaced roughly 250 µm apart, with the entire hippocampus being sampled. Slices were then transferred to a permeabilization solution containing 1.2% Triton-X in PBS for 10 min^34^, before they were incubated in blocking solution containing 15% normal goat serum, 1% bovine serum albumin, and 0.5% Triton-X for 2–4 h at room temperature. Primary antibodies were diluted in this same blocking solution at the following concentrations and incubated at 4 °C for 72 h: rabbit anti-parvalbumin (Swant, PV27; 1:5000) and rabbit anti-prepro-cholecystokinin (Frontier Institute Co., Ab-Rf350; 1:1000). After primary incubation, slices were washed 3 times in PBS for 10 min then incubated in blocking solution containing AlexaFluor488 conjugated secondary antibodies (Abcam, ab150077; 1:1000) for 2–4 h at room temperature. Slices were washed 3 times in PBS for 10 min then incubated in DAPI counterstain (Abcam, ab228549; 2 µM) for 30 min before being washed 3 more times in PBS for 10 min. Slices were mounted on Superfrost Plus slides (Fisher Scientific, 12-550-15) and coverslipped with #1.5 coverslips (Thomas Scientific, 64-0717) using ProLong Gold Antifade mounting media (ThermoFisher, P36930). After mounting media had cured, slides were sealed with CoverGrip (Biotium, 23005).

### Image acquisition and analysis

All GABAergic interneuron marker images were acquired using a Nikon A1R HD25 line-scanning confocal microscope using NIS Elements software. The following argon laser lines were used (in nm): 405 and 488 collected using GaAsP PMTs. All images were a series of z-stacks captured using a piezo motor z-controller, with software set to acquire data in 1024 × 1024 pixel format at a 1/8th frame rate dwell time. One experimenter performed all analyses and was blinded to genotype during acquisition and counting of confocal microscopy data. All GABAergic interneuron marker images were captured using Plan Apo 10 × 0.45 NA objective, with some representative images captured using a Plan Apo 20 × 0.75 NA objective, with images consisting of 11–14 stacks with a z-stack distance of 1 µm and a pinhole size of 19.4 µm. 4–5 hippocampal slices from each animal were imaged across 4 animals per genotype. All images were analyzed in Imaris 9.5 (Bitplane) by manually drawing hippocampal subregion boundaries and hand-counted.

### Statistics and reproducibility

One-way or two-way ANOVA or unpaired, two-tailed Student’s t-test statistical tests were performed where appropriate. For experiments where multiple statistical analyses were performed on the same dataset, our significance threshold was raised to correct for family-wise error rate (FWER) using the Bonferroni post hoc correction method. Sample sizes needed were determined a priori based on power calculations from preliminary data and from previously published data on electrophysiological recordings and immunohistochemical staining. All studies were designed so that an effect size of at least 1 was detected at 80% or greater power, with a minimum sample size of *n* = 7. For all data, replicates are defined as an individual cell or individual slices; no technical replicates were performed. Information on the number of animals used in each experiment can be found in the Supplementary Data File. Statistical tests on electrophysiological recordings were performed on features reported to be under developmental control^40,41^. All statistical analyses were performed in Prism’s GraphPad software and all figures were generated in Adobe’s Illustrator software.

### Reporting summary

Further information on research design is available in the Nature Portfolio Reporting Summary linked to this article.

## Results

### Null *GRIN2A* variants may have a transient seizure susceptibility

**Fig. 1:**
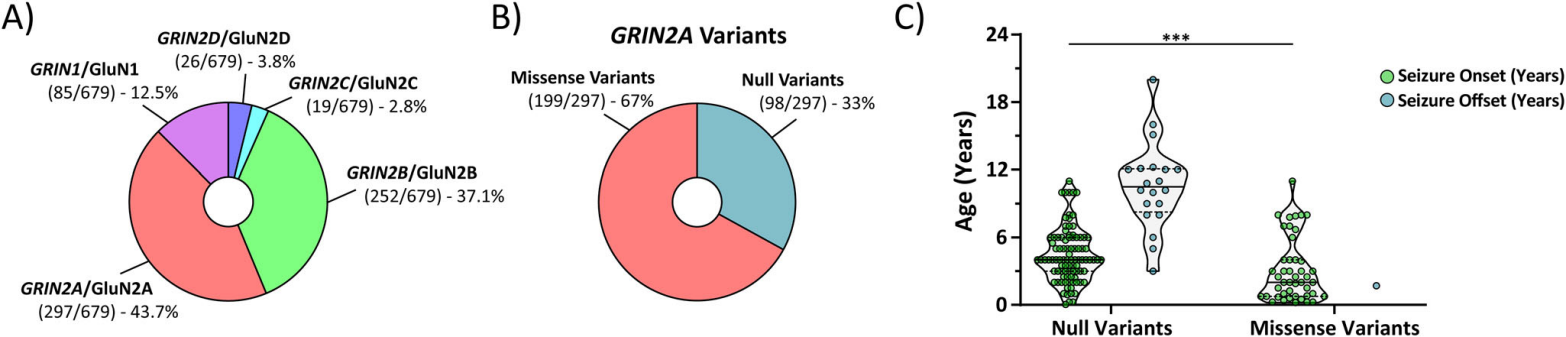
Disease-associated null *GRIN2A* patients may display a transient seizure susceptibility not seen in disease-associated missense *GRIN2A* patients. **A** Summary data adapted from Hansen et al. 2021 showing that most *GRIN* variants are found in the *GRIN2A* (44%; 297/679) gene. **B** Summary data adapted from Hansen et al. 2021 highlighting that a third (98/297) of all *GRIN2A* variants are null variants, which include nonsense variants, as well as chromosomal insertions, deletions, inversions, and translocations. **C** Disease-associated null *GRIN2A* variants display seizure onset susceptibility at a significantly older age than disease-associated missense *GRIN2A* variants (4.5 ± 0.2 years for null *GRIN2A* variants, *n* = 92 vs 3.1 ± 0.4 years for missense *GRIN2A* variants, n = 45; Mann-Whitney ranked sum, *p* = 0.0003). Currently, there are 20 disease-associated null *GRIN2A* patients with a previous history of seizures that were seizure- free at their last follow-up, with a mean seizure offset of 10.4 ± 0.8 years. These data are in stark contrast to disease-associated missense *GRIN2A* variants with available seizure offset data, as only one patient has been reported to be seizure-free at last follow-up (missense *GRIN2A* variant seizure onset age = 0.25 years with seizure offset at 1.7 years). For violin plots, solid middle line represents the median, with the dashed lines representing the 25th and 75th quantile. ***p < 0.001.

Despite strong selective pressure against genetic variation in *GRIN* genes, hundreds of human variants have been reported^1^. Summarizing variant data from Hansen et al. 2021, Fig. 1A highlights that the *GRIN2A* gene contains 44% (297/679) of all known disease-associated *GRIN* variants. Fig. 1B shows that only 67% (199/297) of *GRIN2A* variants are missense, while the remaining 33% (98/297) are null variants as reported in ref. ^1^. Given this striking number of null *GRIN2A* variants, we utilized publicly available patient data (https://grin-portal.broadinstitute.org/) and unpublished clinical data (Supplemental Table S1), to evaluate seizure susceptibility. Figure 1C shows disease-associated null *GRIN2A* variants display seizure onset susceptibility at a significantly older age than disease-associated missense *GRIN2A* variants (4.5 ± 0.2 years for null *GRIN2A* variants, *n* = 92 vs 3.1 ± 0.4 years for missense *GRIN2A* variants, *n* = 45). We also report that 20 null *GRIN2A* patients with previous history of seizures have been seizure-free at last follow-up, with a mean seizure offset of 10.4 ± 0.8 years. These data are in stark contrast to disease-associated missense *GRIN2A* variants with available seizure onset/offset data (Supplemental Table S2). To date, only one disease-associated missense *GRIN2A* patient has been deemed seizure-free at the age of 1.7 years. The mechanism(s) that prevent seizure offset in patients with disease-associated missense *GRIN2A* variants are unknown. Previous data evaluating disease-associated missense *GRIN2A* variants in heterologous systems and knock-in mice show that missense variants have heterogeneous effects, and can alter surface expression, agonist sensitivity, and channel open probability^42,43^. When these variant receptors reach the membrane’s surface, they can trigger direct and persistent effects on synaptic and non-synaptic NMDAR activation^42^. Overall, these data suggest seizure susceptibility in disease-associated null *GRIN2A* patients may be transient, while seizure susceptibility in disease-associated missense *GRIN2A* patients may be sustained. More data is needed to draw conclusions from these findings, including extended periods of follow-up to assess whether any disease-associated null *GRIN2A* patients experience remission from seizure relief. These data do, however, suggest that loss of *GRIN2A* could produce a transient increase in circuit excitability.

### Developing hippocampus shows hyperexcitability in *Grin2a*^+/−^ and *Grin2a*^−/−^ mice

Numerous null variants in *GRIN2A* exist, most of which will prevent or reduce production of mature GluN2A protein as a common feature. Because null variants share reduced expression of GluN2A, *Grin2a*^+/−^ and *Grin2a*^−/−^ mice are an appropriate model that captures this common feature of null variants. Previous data using *Grin2a*^*−/−*^ mice have shown cortical epileptiform activity in preadolescent mice^18^, as well as a prolongation of the NMDAR-mediated excitatory postsynaptic current (EPSC) onto CA1 pyramidal cells^44^ and dentate gyrus granule cells^45^. The prolongation of the NMDAR-mediated EPSC is expected given the mixed expression of rapidly deactivating GluN2A- and slowly deactivating GluN2B-containing NMDARs on excitatory pyramidal cells and the decay kinetics of a pure GluN2B-containing NMDAR population. Moreover, these data suggest that the loss of GluN2A-mediated signaling generates an increased excitability of cortical and hippocampal circuits given the longer time course of the NMDAR-mediated EPSC, however, the exact age range when this excitability change occurs has not been explored. Since GluN2A shows a strong upregulation of expression during early postnatal development^41^, we explored the impact of developmental age on the prolongation of the NMDAR-mediated EPSC.

**Fig. 2:**
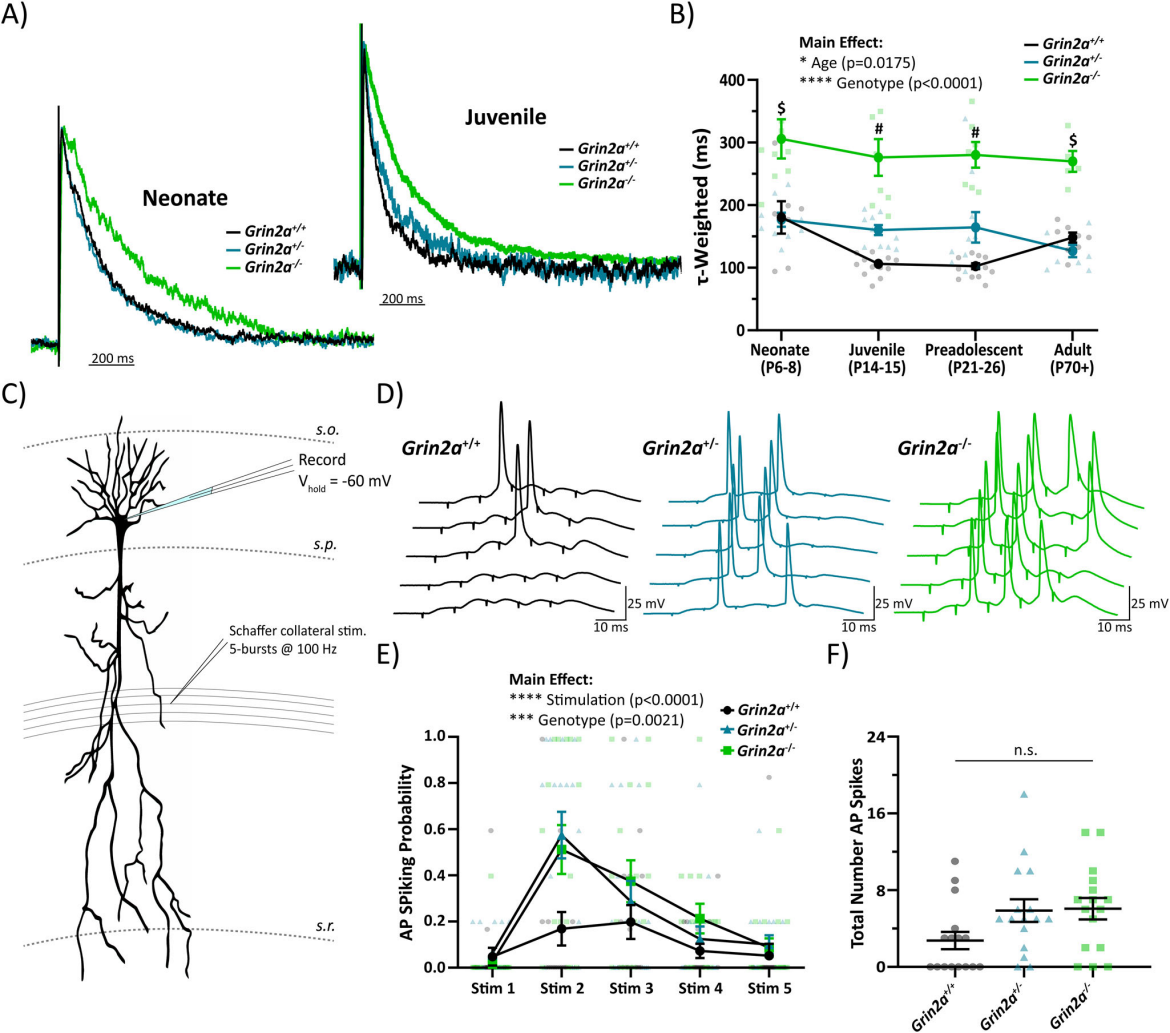
Juvenile CA1 circuit shows hyperexcitability in *Grin2a*^*+/−*^ and *Grin2a*^*−/−*^ mice. **A** Evoked NMDAR-mediated excitatory postsynaptic currents (EPSCs) onto CA1 pyramidal cells from *Grin2a*^*+/+*^*, Grin2a*^*+/−*^, *Grin2a*^*−/−*^ at various ages during development, with normalized representative traces shown for neonate and juvenile mice. **B** Two-way ANOVA showed significant main effects for both age (F_3, 93_ = 3.55; *p* = 0.0175) and genotype (F_3, 93_ = 71.4; *p* < 0.0001) of the tau-weighted describing the NMDAR-mediated EPSC decay time. At both neonate and adult timepoints, only *Grin2a*^*+/+*^ and *Grin2a*^*−/−*^ mice have significantly different NMDAR-mediated EPSC decay times, whereas at the juvenile and preadolescent timepoints, all three genotypes have significantly different NMDAR-mediated EPSC decay times. Given that the juvenile stage was the earliest developmental window with separation at all three genotypes for the NMDAR-mediated EPSC decay time, we conducted further experiments at this age. **C** CA1 pyramidal cells from juvenile mice were current clamped at -60 mV and Schaffer collaterals were stimulated five times at 100 Hz for a total of 5 epochs. Stimulation intensity was set just below threshold to produce an action potential spike after a single Schaffer collateral stimulation. **D** Representative traces showing action potential spiking in response to successive Schaffer collateral stimulations. **E** Action potential spiking probability for each stimulus averaged over 5 epochs across all genotypes. Two-way ANOVA showed significant main effects for both genotype (F_2, 225_ = 6.351; *p* = 0.0021) and stimulation number (F_4, 225_ = 16.82; *p* < 0.0001), however, there was no interaction. **F** Total number of action potentials elicited over 5 epochs of 5-burst Schaffer collateral stimulation. One-way ANOVA showed there was no significant difference across the three genotypes (F = 2.997; *p* = 0.06). Data represented show mean ± SEM. AP = action potential; *s.o*. = *stratum oriens*; *s.p*. = *stratum pyramidale*; *s.r*. = *stratum radiatum*; n.s. = not significant. $ = *Grin2a*^*−/−*^ significantly different than both *Grin2a*^*+/+*^ and *Grin2a*^*+/−*^ at that age; # = all three genotypes significantly different than each other at that age.

Evoked NMDAR-mediated EPSCs were obtained from CA1 pyramidal cells at various ages: neonate (P6-8), juvenile (P14-15), preadolescent (P21-26), and adult (P70+). The weighted tau describing the synaptic deactivation time course of the NMDAR-mediated EPSC was used to assess the relative ratio of GluN2A-containing NMDARs to non GluN2A-containing NMDARs as previous data have shown NMDAR complexes containing the GluN2A subunit have the fastest deactivation time of all four GluN2 subunits, both in heterologous expression systems and in native synapses^1^. The weighted tau of the NMDAR-mediated EPSC in neonatal mice was the most prolonged, regardless of genotype, suggesting very little expression of synaptic GluN2A-containing NMDARs (Fig. 2A, B). The weighted tau measurement in *Grin2a*^*−/−*^ mice was significantly more prolonged at every age when compared to *Grin2a*^*+/+*^ mice (Fig. 2B). These data were expected since CA1 pyramidal cells have been shown to express a mixture of GluN2A-containing and GluN2B-containing NMDARs^46^, with the presumption that GluN2B-containing NMDARs will be the only subunit present at the CA1 pyramidal cell synapse in the *Grin2a*^*−/−*^ mouse. The weighted tau of the NMDAR-mediated EPSCs in juvenile and preadolescent mice were significantly different across all three genotypes, with each following a *Grin2a* gene dosing-dependent shortening of weighted tau (Fig. 2B). In adult mice, the weighted tau measurements in *Grin2a*^*+/+*^ and *Grin2a*^*+/−*^ mice overlap, suggesting that *Grin2a*^*+/−*^ mice eventually reach a wild-type weighted tau. The increased weighted tau between the preadolescent and adult timepoints in *Grin2a*^*+/+*^ mice was unexpected and could be due to an increased insertion of GluN2B-containing NMDARs in adult mice after the preadolescent critical period of plasticity and development. More importantly, these data highlight that the juvenile developmental timepoint may be the start of aberrant synaptic signaling, at least in terms of charge transfer and calcium influx.

Since the earliest divergence in NMDAR-mediated EPSC weighted tau across all three genotypes occurred in juvenile mice, this age was our benchmark to explore what other changes to hippocampal circuit function, if any, occur in *Grin2a*^*+/−*^ and *Grin2a*^*−/−*^ mice. Thus, we recorded action potential generation probability in juvenile CA1 pyramidal cells during five Schaffer collateral stimulations at 100 Hz (e.g. a 50 ms burst of 5 pulses) to assess circuit output after excitatory afferent signaling (Fig. 2C). Two-way ANOVA shows a statistically significant main effect for both stimulation number and genotype (Fig. 2D, E; Supplemental Table S4), however, there are no significant interactions nor differences in the total number of action potentials generated (Fig. 2E, F; Supplemental Table S5). These data suggest that *Grin2a*^*+/−*^ and *Grin2a*^*−/−*^ mice are more likely to fire action potentials when subjected to similar excitatory afferent activity compared to *Grin2a*^*+/+*^ mice.

One possible interpretation of these data is that the loss of *Grin2a* triggers a compensatory upregulation of other NMDAR subunits, especially GluN2B. Data from whole hippocampus mRNA screening indicates no significant differences in the *Grin1*, *Grin2b*, and *Grin2c* genes across all three genotypes of juvenile mice (Supplemental Fig. S1). mRNA data do show a statistically significant upregulation of both *Grin2d* and *Grin3a*, but these upregulations only occur in *Grin2a*^*−/−*^ mice (Supplemental Fig. S1). Since our synaptic spiking data show similar hyperexcitability in both *Grin2a*^*+/−*^ and *Grin2a*^*−/−*^ mice, these data are likely not explanatory for our observed spiking phenotype. Alternatively, heightened synaptic spiking could be due to alterations in the intrinsic excitability of the CA1 pyramidal cells themselves. We show, however, no differences in any measurable electrophysiological passive properties such as resting membrane potential or input resistance, as well as no changes in action potential characteristics or firing properties (Supplemental Fig. S2; Supplemental Table S5). Thus, changes in action potential firing probability in *Grin2a*^*+/−*^ and *Grin2a*^*−/−*^ mice are not due to alterations in NMDAR subunit mRNA expression or in CA1 pyramidal cell intrinsic excitability. In addition to providing direct excitatory afferent signaling onto pyramidal cells, Schaffer collateral stimulation will also provide excitatory tone onto feedforward GABAergic interneurons. In addition, we show that the total loss of the *Grin2a* gene promotes the upregulation of *Grin2d* mRNA, which has been found to be exclusive to GABAergic interneurons in the hippocampus^29,47^. For these reasons, we chose to further explore the GABAergic interneuron network in developing CA1.

### Alterations in hippocampal PV cell density

Alterations in synaptic excitability as described Fig. 2 could be due to changes in several features of the hippocampal circuit, including GABAergic inhibition which is mediated by a wide range of different interneurons. Inhibitory GABAergic basket cells primarily make somatic inhibitory synaptic connections where they exert profound control on pyramidal cell firing^48^, and in CA1, are heavily innervated by Schaffer collateral afferents^49^. CA1 basket cells can be split into two main subtypes: parvalbumin (PV)-expressing cells and cholecystokinin (CCK)-expressing cells^49^. PV and CCK cells have opposing transcriptomic profiles, and each represents a major subtype of interneuron arising from the medial ganglionic eminence (MGE) and caudal ganglionic eminence (CGE), respectively^49^. Since early pyramidal cell activity has been shown to control interneuron death^50^, and we report alterations to the NMDAR-mediated EPSC onto young pyramidal cells, we hypothesized that the loss of *Grin2a* may impact basket cell density in CA1. We therefore determined cell density of PV and CCK interneurons in *Grin2a*^+/+^, *Grin2a*^+/−^, and *Grin2a*^−/−^ mice via immunohistochemical staining.

**Fig. 3:**
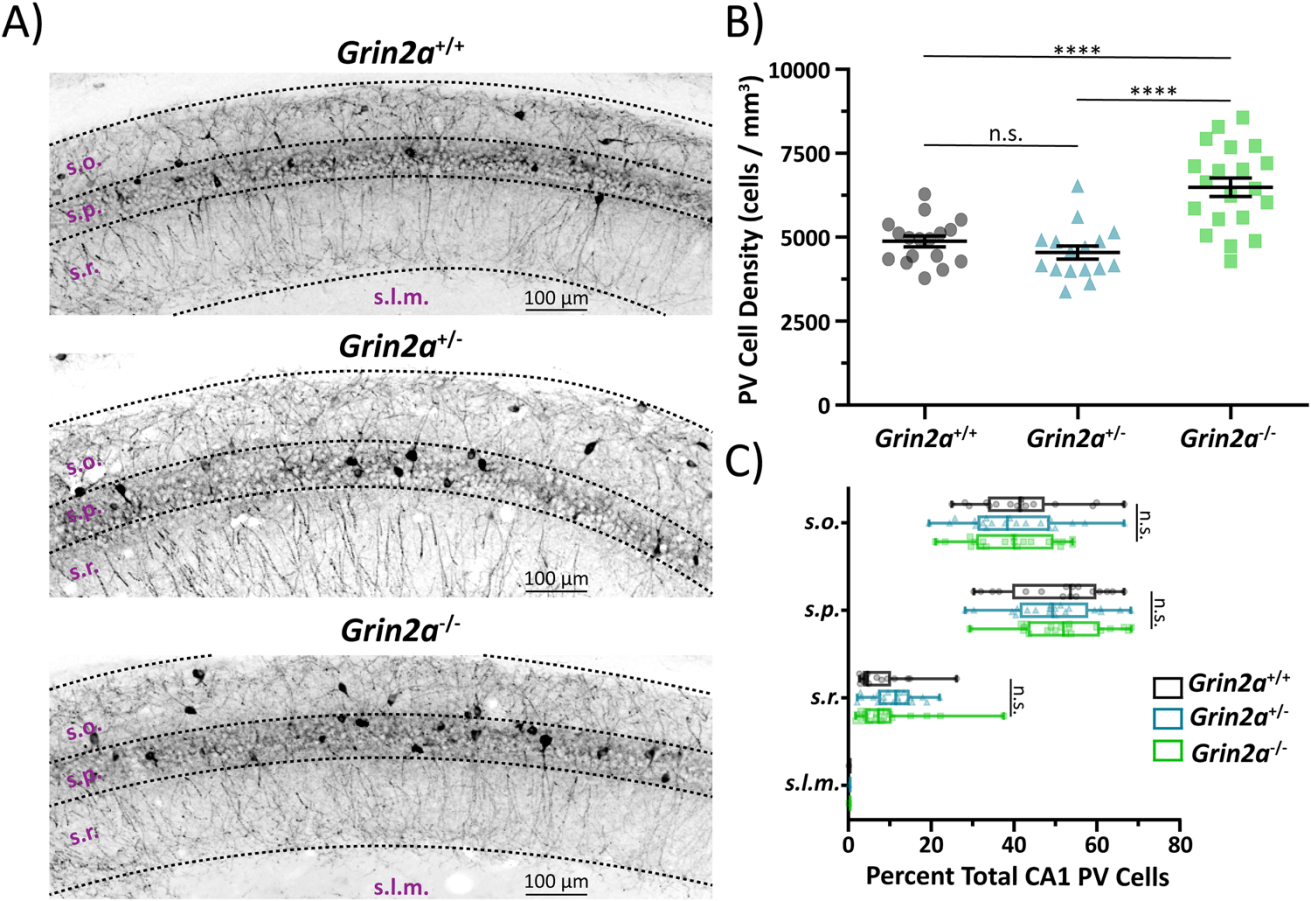
Loss of *Grin2a* causes an increase in parvalbumin (PV) cell density in CA1. **A** Representative images of CA1 hippocampal sections stained for PV in preadolescent mice. **B** CA1 PV cell density is significantly increased in *Grin2a*^−/−^ mice compared to *Grin2a*^+/+^ mice (6488 ± 276 cells per mm^3^ in *Grin2a*^−/−^ vs 4875 ± 162 cells per mm^3^ in *Grin2a*^+/+^; one-way ANOVA, *p* < 0.0001) and *Grin2a*^+/−^ mice (6488 ± 276 cells per mm^3^ in *Grin2a*^−/−^ vs 4542 ± 198 cells per mm^3^ in *Grin2a*^+/−^; one-way ANOVA, *p* < 0.0001). There is no difference in CA1 PV cell density between *Grin2a*^+/+^ and *Grin2a*^+/−^ mice (one-way ANOVA, *p* = 0.58). **C** Despite an increase in cell density in *Grin2a*^−/−^ mice, there is no difference in PV CA1 cellular lamination across all three genotypes. Data represented show mean ± SEM. *s.o*. = *stratum oriens*; *s.p*. = *stratum pyramidale*; *s.r*. = *stratum radiatum*; *s.l.m*. = *stratum lacunosum moleculare*; PV = parvalbumin; ****p < 0.0001; n.s. = not significant.

**Fig. 4:**
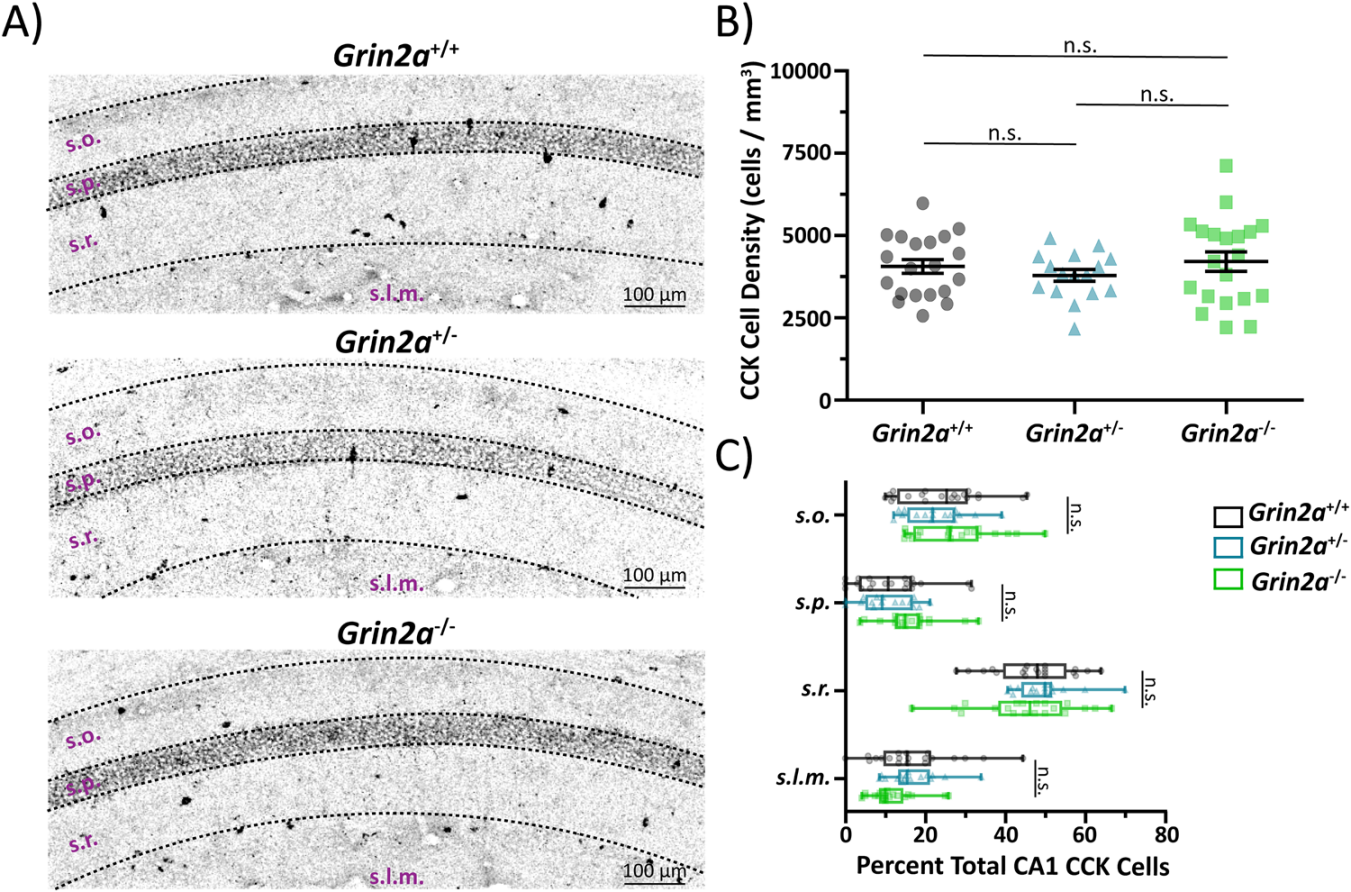
Loss of *Grin2a* does not alter cholecystokinin (CCK) cell density in CA1. **A** Representative images of CA1 hippocampal sections stained for CCK in preadolescent mice. **B** CA1 CCK cell density is unchanged across all three genotypes (one-way ANOVA, *p* = 0.49). **C** There is no difference in CCK CA1 cellular lamination across all three genotypes. Data represented show mean ± SEM. *s.o*. = *stratum oriens*; *s.p*. = *stratum pyramidale*; *s.r*. = *stratum radiatum*; *s.l.m*. = *stratum lacunosum moleculare*; CCK = cholecystokinin; n.s. = not significant.

The total loss of *Grin2a* promotes an increase in the total number of PV cells in CA1 (Fig. 3A, B; Supplemental Table S6) of preadolescent mice compared to both *Grin2a*^+/+^ and *Grin2a*^+/−^. This observed increase in CA1 PV cells in *Grin2a*^−/−^ mice also remained into adulthood (Supplemental Fig. S3). Although the overall cell density of PV cells is altered in *Grin2a*^−/−^ mice, there is no difference in PV cellular lamination (Fig. 3A, C; Table Supplemental S6) regardless of genotype, with most PV cells residing in *stratum oriens* and *stratum pyramidale*^49^. Unlike PV cells, we show that the loss of *Grin2a* does not impact CCK cell density (Fig. 4A, B**;** Table Supplemental S7) or CCK cellular lamination (Fig. 4A, C; Supplemental Table S7). Given that we observed an increase in CA1 PV cell density in *Grin2a*^*−/−*^ mice, we also wanted to check if these extra PV cells showed any morphological differences from age-matched CA1 PV cells from *Grin2a*^*+/+*^ mice. Using biocytin-backfilled reconstructions of preadolescent *Grin2a*^*+/+*^ and *Grin2a*^*−/−*^ CA1 PV cells, we report no change in dendritic/axonal morphology and laminar targeting (Supplemental Fig. S4). Thus, these data indicate that the loss of *Grin2a* may preferentially impact PV interneuron survival/death in CA1. Moreover, the effect of a detectable increased cell density in only *Grin2a*^−/−^ mice suggests that a threshold of aberrant pyramidal cell activity must be reached before PV cell survival/death is impacted, with little to no impact on cellular morphology or neurite targeting. Pyramidal cell activity thus far has only been shown to impact MGE-derived interneurons, with no data on survival/apoptosis of CGE-derived interneurons^50^. Moreover, CCK cells may not be affected since previous reports have shown that these cells have little to no GluN2A-mediated synaptic signaling^44,51^. Although a change in the PV cell density will likely contribute to aberrant CA1 circuit function, these data alone are unlikely to explain our observed action potential spiking phenotype.

### Age-dependent changes in passive and action potential firing properties of CA1 PV cells

In addition to controlling interneuron survival/death, local pyramidal cell activity has also been implicated in controlling the maturation of GABAergic interneurons^50,52^. Previous work has shown that PV cells undergo an electrophysiological maturation of both their passive and action potential firing properties, however, many of these studies have been performed in cortical PV cells^40,53,54^ and dentate gyrus PV cells^55^. Since clear data highlighting an electrophysiological maturation in CA1 is limited (however, see ref. ^36^), we first wanted to demonstrate that hippocampal PV cells also show an electrophysiological maturation pattern like those seen in cortex.

Detailed analysis of the electrophysiological maturation pattern in young PV cells has been hampered by the age-dependent expression of the PV gene itself, which begins around P14^56^. To bypass this limitation, we used another driver mouse line, *Tac1*-Cre (Jax #:021877), to obtain neonatal (P6-8) PV cells recordings in developing hippocampus. *Tac1* is highly expressed in PV cells, with little to no expression reported in MGE-derived somatostatin cells^36,37^. In addition, *Tac1* expression begins early in embryogenesis and is sustained well into adulthood (Allen Mouse Brain Atlas). To confirm that *Tac1*-Cre mice are a viable tool for studying neonatal PV cells, we first performed immunohistochemical colocalization staining to confirm that PV cells do indeed express the *Tac1* gene. Adult tissue from *Tac1*-Cre x Floxed-eGFP mice were stained for eGFP and PV, with data reporting that 90 ± 5% of all PV-positive cells also stained positive for eGFP (Supplemental Fig. S5), whereas 52 ± 3% of eGFP positive cells also stained positive for PV (Supplemental Fig. S5). Closer evaluation of these data reveal that the majority of PV/eGFP overlap occurs in the *stratum oriens* and *stratum pyramidale* layers of CA1. For these reasons, only *Tac1*-positive cells from these two laminae were chosen for recording. Moreover, all patch-clamped cells using *Tac1*-Cre x Floxed-eGFP mice were biocytin-backfilled and visually confirmed to have no dendritic spines, as some *Tac1*-positive puncta appear to be pyramidal cells (Supplemental Fig. S6). We also compared several passive and action potential firing properties in juvenile (P14-15) *Tac1*-Cre positive cells against a traditional *Pvalb*-TdTomato driver line (Jax #:027395) and found no differences (Supplemental Fig. S6).

**Fig. 5:**
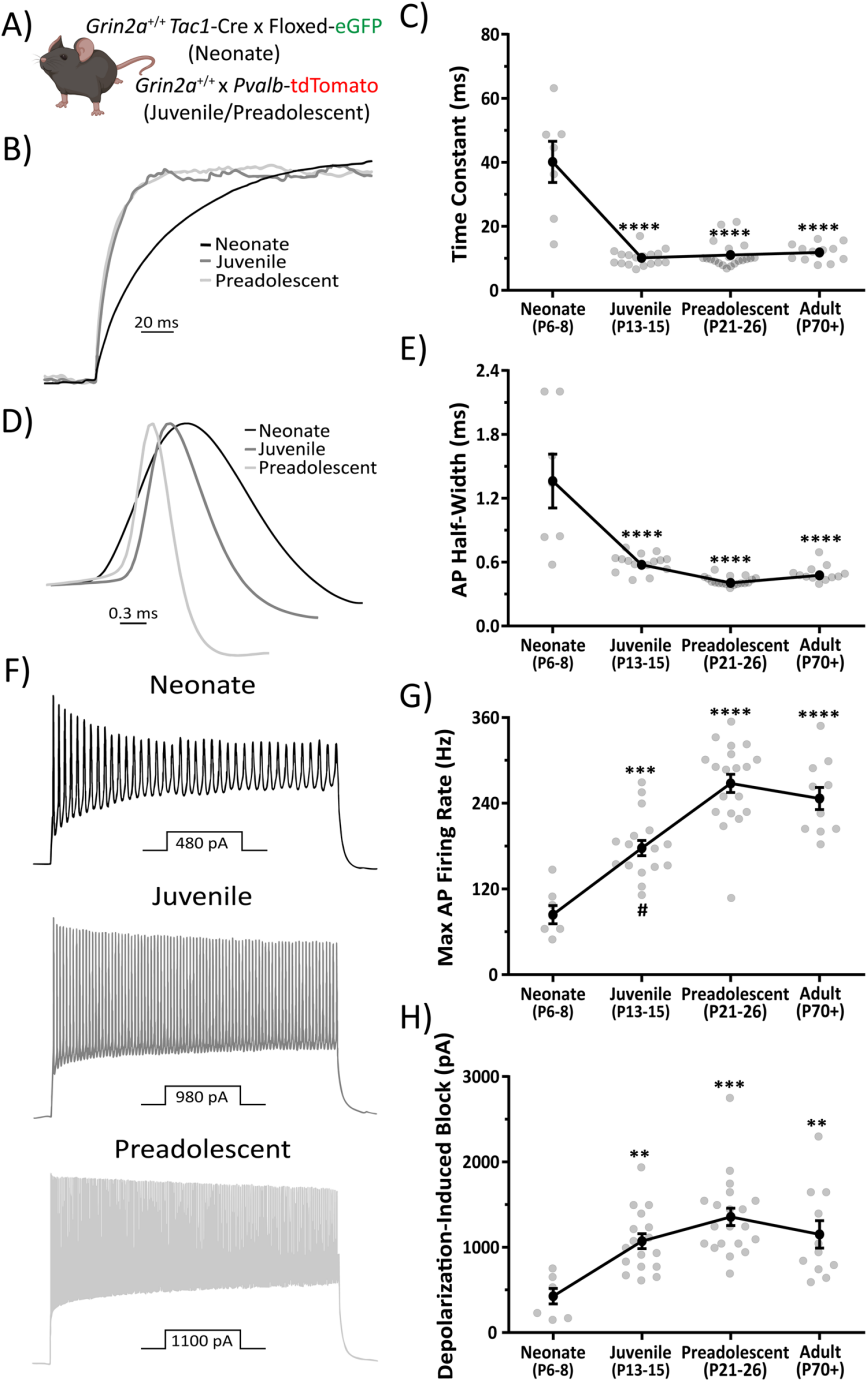
CA1 PV cells undergo electrophysiological maturation of passive and action potential firing properties. **A** PV cells were visualized using either *Pvalb*-TdTomato or *Tac1*-Cre x floxed eGFP (see Supplemental Figs. S5 and S6). Mouse clipart from BioRender (permissions provided). **B** Representative, amplitude-normalized repolarization traces to highlight differences in membrane time constant following a −50 pA current injection at different developmental timepoints. **C** Membrane time constant is significantly prolonged in neonatal mice (F = 40.68, one-way ANOVA; *p* < 0.0001 for post-hoc multiple comparisons with every other developmental timepoint). **D** Action potential half-width is significantly prolonged in neonatal mice (F = 28, one-way ANOVA; *p* < 0.0001 for post-hoc multiple comparisons with every other developmental timepoint). **E** Representative, amplitude-normalized single action potential traces to highlight differences in half-width at different developmental timepoints. **F** Representative action potential trains elicited by various current injections depicted below each train to illustrate change in maximum action potential firing frequency during development. Traces shown are those just below threshold for depolarization-induced block of action potential firing. **G** Maximum action potential firing frequency is significantly decreased in neonatal mice (F = 28.3, one-way ANOVA; *p* < 0.001 for post-hoc multiple comparison test with juvenile mice, and *p* < 0.0001 for post-hoc multiple comparison test with preadolescent and adult mice). Juvenile mice also show a significantly decreased maximum action potential firing frequency compared to preadolescent mice (one-way ANOVA post-hoc multiple comparison test; *p* = 0.0039). **H** Current required for depolarization-induced block of action potential firing is significantly decreased in neonatal mice (F = 8.36, one-way ANOVA; *p* < 0.01 for post-hoc multiple comparison test with juvenile and adult mice, and *p* < 0.0001 for post-hoc multiple comparison test with preadolescent mice). Symbols are mean ± SEM. AP = action potential; depolarization block = Current required for depolarization-induced block of action potential firing. **p < 0.01; ***p < 0.001; ****p < 0.0001; # = juvenile mice significantly different than adult mice in one-way ANOVA post-hoc multiple comparison test.

Using *Tac1*-Cre mice for neonatal recordings and *Pvalb*-TdTomato mice for juvenile, preadolescent, and adult recordings we assayed various passive and action potential firing properties of CA1 PV cells at four stages during neurodevelopment (Fig. 5A). We report that neonatal CA1 PV cells show a transient prolongation of their membrane time constant, which is significantly increased compared to juvenile, preadolescent, and adult CA1 PV cells (Fig. 5B, C; Supplemental Table S8). A similar trend is observed for input resistance in neonatal CA1 PV Cells (Supplemental Table S8). The action potential half-width of neonatal CA1 PV cells is also transiently prolonged and is significantly increased compared to juvenile, preadolescent, and adult cells (Fig. 5D, E; Supplemental Table S8). A transient prolongation of the membrane time constant and action potential half-width observed here matches previously reported data for developing cortical PV cells^40^. We also report dampening of the maximum action potential firing frequency in developing CA1 PV cells. Neonatal CA1 PV cell maximum action potential firing capacity is significantly decreased compared to juvenile, preadolescent, and adult CA1 PV cells (Fig. 5F, G; Supplemental Table S8). The maximum action potential firing frequency in juvenile CA1 PV cells is also significantly decreased compared to adult cells (Fig. 5F, G; Supplemental Table S8). An age-dependent increase in the fast-spiking nature of PV cells has been well characterized and is thought to be driven by a delay in the upregulation of the rapid Kv3-family of voltage-gated potassium channels^40^. The current required for depolarization-induced block of action potential firing in neonatal CA1 PV cells is significantly decreased compared to juvenile, preadolescent, and adult cells (Fig. 5F, H; Supplemental Table S8). Here, the decreased current for depolarization-induced block is likely caused by an increased input resistance measured in neonatal mice. In all, we show that CA1 PV cells do undergo significant electrophysiological maturation programs, transforming them from simple signal propagators to precise signaling processors.

### Electrophysiological maturation of PV cells is delayed in *Grin2a*^+/−^ and *Grin2a*^−/−^ mice

**Fig. 6:**
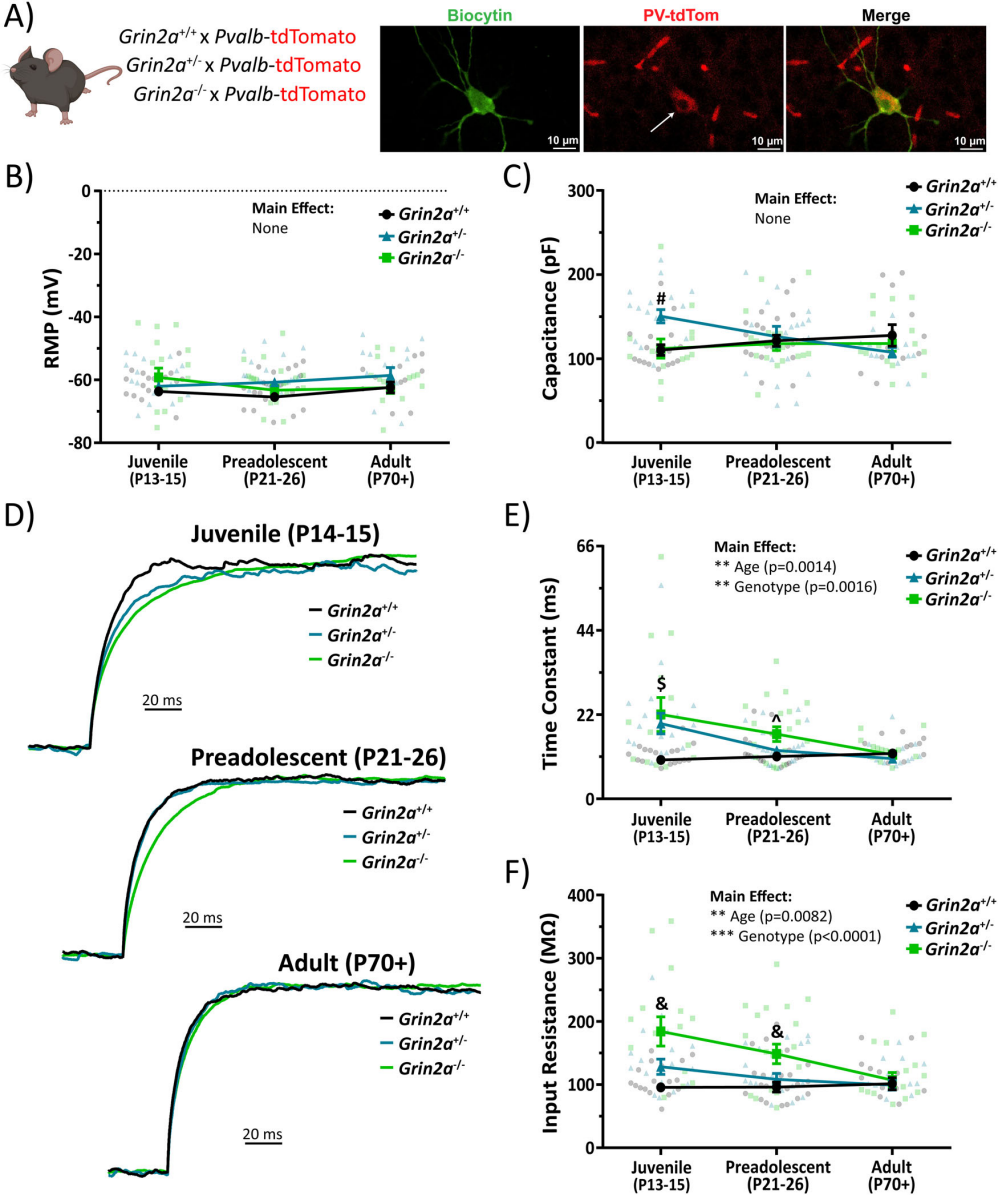
The loss of *Grin2a* causes a transient change in passive electrophysiological properties in CA1 PV cells. **A** Mouse model used to visualize PV cells in CA1 next to examples of a biocytin backfilled CA1 PV cell that was stained for TdTomato to indicate successful cellular identification. Mouse clipart from BioRender (permissions provided). **B** No change in resting membrane potential across all three genotypes at different developmental timepoints (two-way ANOVA). **C** No main effect of cellular capacitance across genotype (F_2, 136_ = 1.3; *p* = 0.28; two-way ANOVA) or age (F_2, 136_ = 0.32; *p* = 0.72; two-way ANOVA), however, there is a statistically significant interaction such that juvenile *Grin2a*^*+/−*^ mice have a higher cellular capacitance than both *Grin2a*^*+/+*^ mice (150 ± 8.0 pF for *Grin2a*^*+/−*^ vs 110 ± 6.5 pF for *Grin2a*^*+/+*^; *p* = 0.0061) and *Grin2a*^*−/−*^ mice (150 ± 8.0 pF for *Grin2a*^*+/−*^ vs 111 ± 11 pF for *Grin2a*^*−/−*^; *p* = 0.014). **D** Representative, amplitude-normalized repolarization traces to highlight differences in membrane time constant following a −50 pA current injection at different developmental timepoints. **E** Membrane time constant measurements show statistically significant main effects for both age (F_2, 136_ = 6.9; *p* = 0.0014; two-way ANOVA) and genotype (F_2, 136_ = 6.8; *p* = 0.0016; two-way ANOVA). There are also several statistically significant interactions such that both juvenile *Grin2a*^*+/−*^ mice (20 ± 2.7 ms for *Grin2a*^*+/−*^ vs 10 ± 0.6 ms for *Grin2a*^*+/+*^; *p* = 0.0011) and *Grin2a*^*−/−*^ mice (22 ± 4.4 ms for *Grin2a*^*−/−*^ vs 10 ± 0.6 ms for *Grin2a*^*+/+*^; *p* < 0.0001) displayed higher membrane time constants than *Grin2a*^*+/+*^ mice. In addition, preadolescent *Grin2a*^*−/−*^ mice showed a higher membrane time constant than *Grin2a*^*+/+*^ mice (17 *±* 1.9 ms for *Grin2a*^*−/−*^ vs 11 ± 0.9 ms for *Grin2a*^*+/+*^; *p* = 0.048). **F** Input resistance measurements show statistically significant main effects for both age (F_2, 133_ = 5.0; *p* = 0.0082; two-way ANOVA) and genotype (F_2, 133_ = 12.2; *p* < 0.0001; two-way ANOVA). There are also several statistically significant interactions such that juvenile *Grin2a*^*−/−*^ mice displayed higher input resistances than *Grin2a*^*+/+*^ mice (184 ± 23 MΩ for *Grin2a*^*−/−*^ vs 96 ± 6.1 MΩ for *Grin2a*^*+/+*^; *p* < 0.0001) and *Grin2a*^*+/−*^ mice (184 ± 23 MΩ for *Grin2a*^*−/−*^ vs 128 ± 12 MΩ for *Grin2a*^*+/+*^; *p* = 0.0054). In addition, preadolescent *Grin2a*^*−/−*^ mice showed a higher input resistance than *Grin2a*^*+/+*^ mice (149 ± 15 MΩ for *Grin2a*^*−/−*^ vs 96 ± 7.9 MΩ for *Grin2a*^*+/+*^; *p* = 0.0031) and *Grin2a*^*+/−*^ mice (149 ± 15 MΩ for *Grin2a*^*−/−*^ vs 108 ± 9.3 MΩ for *Grin2a*^*+/+*^; *p* = 0.043). The sum of these data indicates an age- and gene-dependent transient delay in various passive electrical properties of CA1 PV cells. Symbols are mean ± SEM. RMP = resting membrane potential; # = *Grin2a*^*+/−*^ significantly different than both *Grin2a*^*+/+*^ and *Grin2a*^*−/−*^ at that age; $ *=* *Grin2a*^*+/+*^ significantly different than both *Grin2a*^*+/−*^ and *Grin2a*^*−/−*^ at that age; ^ *=* *Grin2a*^*−/−*^ significantly different than *Grin2a*^*+/+*^ at that age; & = *Grin2a*^*−/−*^ significantly different than both *Grin2a*^*+/+*^ and *Grin2a*^*+/−*^ at that age; **p < 0.01; ***p < 0.001.

After showing that CA1 PV cells in *Grin2a*^*+/+*^ mice undergo electrophysiological maturation of their passive and action potential firing properties, we tested our hypothesis that altered network activity may impact the rate of PV cell maturation in *Grin2a*^*+/−*^ and *Grin2a*^*−/−*^ mice. We generated *Grin2a*^*+/+*^:*Pvalb*-tdTom, *Grin2a*^*+/−*^:*Pvalb*-tdTom, and *Grin2a*^*−/−*^:*Pvalb*-tdTom mice via selective breeding (see methods) to visualize PV cells in CA1 across these three genotypes (Fig. 6A). We found no difference in the resting membrane potential of juvenile, preadolescent, or adult CA1 PV cells in *Grin2a*^*+/+*^, *Grin2a*^*+/−*^, and *Grin2a*^*−/−*^ mice (Fig. 6B; Supplemental Table S9). We did not find any statistically significant main effects of age or genotype for PV cellular capacitance (Fig. 6C; Supplemental Table S9). Both the membrane time constant and input resistance both had statistically significant main effects for age and genotype, indicating a transient increase in both measures of passive membrane excitability (Fig. 6E, F; Supplemental Table S9). Moreover, we found statistically significant interactions for membrane time constant values indicating that membrane time constant decreases in an age- and *Grin2a* gene-dosing dependent manner (Fig. 6D, E; Supplemental Table S9). Overall, we found that *Grin2a*^*+/−*^ mice didn’t reach *Grin2a*^*+/+*^ membrane time constant values until preadolescence, while *Grin2a*^*−/−*^ mice didn’t reach *Grin2a*^*+/+*^ membrane time constant values until adulthood. Importantly, though, all membrane time constant values eventually did attain *Grin2a*^*+/+*^ levels (Fig. 6D, E; Supplemental Table S9). We saw this same trend for input resistance values (Fig. 6F; Supplemental Table S9). The sum of these data illustrates that there is an age- and genotype-dependent transient delay in the passive membrane excitability of CA1 PV cells. That is, values reach those of *Grin2a*^*+/+*^ eventually, but remain at abnormal levels for an extended period that is dependent on the number of functional copies of *Grin2a*.

**Fig. 7:**
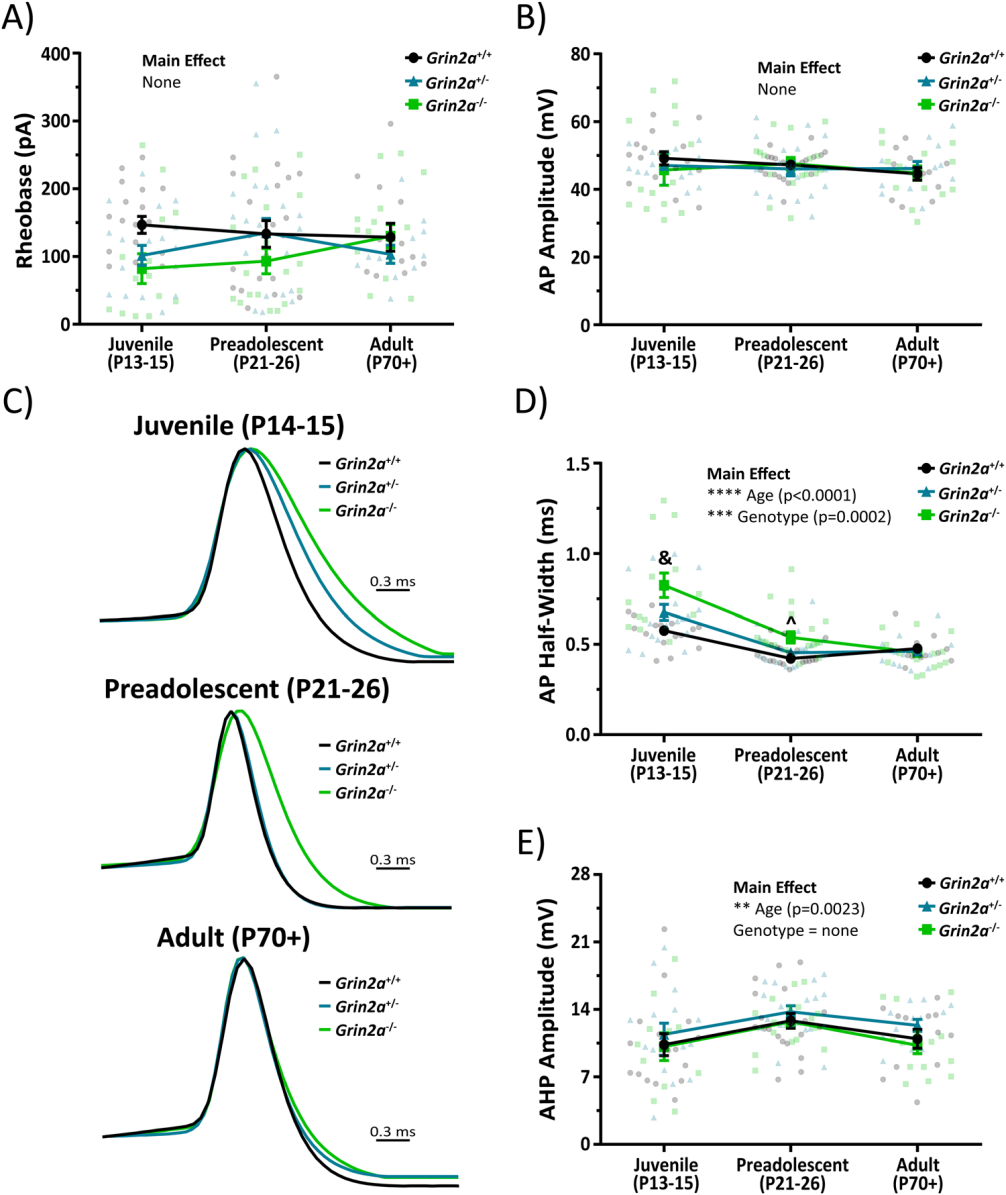
The loss of *Grin2a* causes a transient change in action potential waveform properties of CA1 PV cells. There are no differences in **A** rheobase or **B** action potential amplitude regardless of age or genotype in CA1 PV cells. **C** Representative, amplitude-normalized single action potential traces to highlight differences in half-width at different developmental timepoints. **D** Action potential half-width measurements show statistically significant main effects for both age (F_2, 134_ = 47; *p* < 0.0001; two-way ANOVA) and genotype (F_2, 134_ = 9.3; *p* = 0.0002; two-way ANOVA). There are also several statistically significant interactions such that both juvenile *Grin2a*^*+/+*^ mice (0.58 ± 0.02 ms for *Grin2a*^*+/+*^ vs 0.82 *±* 0.07 ms for *Grin2a*^*−/−*^; *p* < 0.0001) and *Grin2a*^*+/−*^ mice (0.68 ± 0.04 ms for *Grin2a*^*+/−*^ vs 0.82 ± 0.07 for *Grin2a*^*−/−*^; *p* < 0.005) displayed longer action potential half-widths than *Grin2a*^*−/−*^ mice. In addition, preadolescent *Grin2a*^*−/−*^ mice showed longer action potential half-widths than *Grin2a*^*+/+*^ mice (0.54 ± 0.03 ms for *Grin2a*^*−/−*^ vs 0.42 ± 0.01 ms for *Grin2a*^*+/+*^; *p* = 0.0152). **E** Afterhyperpolarization amplitude of the action potential waveform showed a significant main effect for age (F_2, 127_ = 6.36; *p* = 0.0023; two-way ANOVA), but no main effect for genotype (F_2, 127_ = 2; *p* = 0.14; two-way ANOVA). Symbols are mean ± SEM. AP = action potential; AHP = afterhyperpolarization; ^ = *Grin2a*^*−/−*^ significantly different than *Grin2a*^*+/+*^ at that age; & = *Grin2a*^*−/−*^ significantly different than both *Grin2a*^*+/+*^ and *Grin2a*^*+/−*^ at that age; **p < 0.01; ***p < 0.001; ****p < 0.0001.

**Fig. 8:**
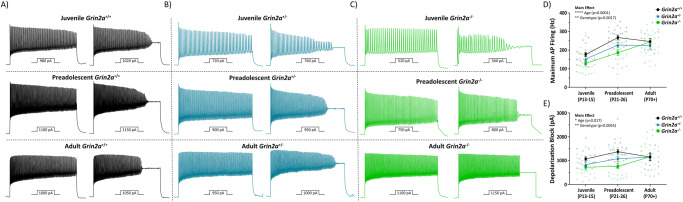
The loss of *Grin2a* causes a transient change in action potential firing properties of CA1 PV cells. Representative action potential trains elicited by current injections depicted below each train to illustrate the maximum action potential firing frequency and the current required for depolarization-induced block of action potential firing for **A** *Grin2a*^*+/+*^, **B** *Grin2a*^*+/−*^, and **C** *Grin2a*^*−/−*^ mice during development. **D** Maximum action potential firing frequencies show a significant main effect for both age (F_2, 135_ = 29.8; *p* < 0.0001; two-way ANOVA) and genotype (F_2, 135_ = 6.7; *p* = 0.0017; two-way ANOVA). **E** Currents required to reach depolarization-induced block of action potential firing show a significant main effect for both age (F_2, 135_ = 4.2; *p* = 0.017; two-way ANOVA) and genotype (F_2, 135_ = 5.4; *p* = 0.006; two-way ANOVA). Symbols are mean ± SEM. AP = action potential; depolarization block = current required to reach depolarization-induced block of action potential firing; *p < 0.05; **p < 0.01; ***p < 0.001; ****p < 0.0001.

We also examined the action potential waveform and firing properties of CA1 PV cells in *Grin2a*^*+/−*^ and *Grin2a*^*−/−*^ mice during development. We show no statistically significant effects of age or genotype on rheobase or action potential amplitude, as well as no significant interactions for each measure (Fig. 7A, B; Supplemental Table S10). Action potential half-width, however, shows statistically significant effects for both age and genotype (Fig. 7C, D; Supplemental Table S10). Statistically significant interactions for the action potential half-width indicate that it decreases in an age- and *Grin2a* gene-dosing dependent manner (Fig. 7C, D; Supplemental Table S10). Overall, we found that *Grin2a*^*+/−*^ mice didn’t reach *Grin2a*^*+/+*^ action potential half-width values until preadolescence, while *Grin2a*^*−/−*^ mice didn’t reach *Grin2a*^*+/+*^ action potential half-width values until adulthood. Importantly, though, all action potential half-width values eventually did attain *Grin2a*^*+/+*^ levels (Fig. 7D, E; Supplemental Table S10). These data indicate a significant but transient prolongation of the action potential half-width that is dependent on both age and genotype, in line with data obtained on the membrane time constant and input resistance. The afterhyperpolarization amplitude shows a statistically significant main effect for age, but not for genotype, which suggests that the channels responsible for afterhyperpolarization amplitude are not subjected to the same delay observed for other measures like membrane time constant, input resistance, and action potential half-width (Fig. 7E; Supplemental Table S10). We also found that both the maximum action potential firing frequency before depolarization-induced block and the current required to reach depolarization-induced block of action potential firing show statistically significant main effects for age and genotype (Fig. 8A-E; Supplemental Table S10).

The sum of these results suggests that the loss of *Grin2a* impacts the electrophysiological maturation programs of CA1 PV cells, however, this delay is only transient as all perturbed measures eventually attain *Grin2a*^*+/+*^ levels. Immature PV cells may be fast to fire given their increased input resistance, but they are also fast to retire given their decreased threshold for depolarization-induced block and diminished action potential firing frequency. That is, inhibitory firing will fade in response to high frequency firing, as might occur during initial runup toward seizure initiation. Diminished inhibitory output of a prominent interneuron subtype (PV) could have profound consequences on circuit excitability and network wiring during the critical plasticity period of preadolescent development. These alterations in circuit function could then manifest as epileptiform activity, maladaptive plasticity, or cognitive impairment. Moreover, if the transient nature of these reported alterations in PV cell function also occur in PV interneurons from *GRIN2A* null variant patients, this could provide a molecular mechanism driving hyperexcitability and hypersynchronous epileptiform activity in these patients.

## Discussion

The most important finding of this study is that disease-associated null *GRIN2A* human patients display a largely transient seizure susceptibility that resolves with age. To explore this new clinical finding at the circuit and cellular level, we conducted studies using *Grin2a*^*+/−*^ and *Grin2a*^*−/−*^ mice at various stages during neurodevelopment. While the use of the *Grin2a*^−/−^ mice is not an exact model of any individual disease-associated *GRIN2A* null variant, it captured a shared feature of most null variants, which is reduced GluN2A expression. These mice were generated via insertion of translation-interrupting sequences within the coding region of the transmembrane domain (see “Methods”), meaning like many null variants, they will generate the initial portion of the polypeptide chain. By utilizing the weighted tau of evoked NMDAR-mediated EPSCs from *Grin2a*^*+/+*^, *Grin2a*^*+/−*^ and *Grin2a*^*−/−*^ mice we determined that the juvenile (P14-15) time window was the earliest neurodevelopmental stage in which loss of GluN2A-mediated signaling would impart increased circuit excitability in terms of overall charge transfer. Here, the loss of GluN2A-containing NMDARs, which have the fastest deactivation kinetics of all four GluN2 subunits^1^, will likely increase the excitatory tone onto every neuron that typically expresses GluN2A. Moreover, we show using a multiple stimulus action potential spiking paradigm, increased circuit excitability and CA1 pyramidal cell output in juvenile mice of both *Grin2a*^*+/−*^ and *Grin2a*^*−/−*^ mice. These alterations in somatic spiking are not due to global upregulation of most *Grin* genes (including *Grin2b*) nor can they be attributed to perturbations in the intrinsic excitability or action potential firing properties of CA1 pyramidal cells.

Deeper evaluation of the developing CA1 circuit led us to uncover age- and *Grin2a* gene dosing-dependent transient delays in the electrophysiological maturation programs of PV interneurons. Overall, we report that *Grin2a*^*+/+*^ mice reach electrophysiological maturation between the neonatal and juvenile neurodevelopmental timepoints (except for maximum action potential firing frequency), with *Grin2a*^*+/−*^ mice not reaching electrophysiological maturation until preadolescence, and *Grin2a*^*−/−*^ not reaching electrophysiological maturation until adulthood. The significance of these findings are multifaceted, with the most important being that these data may represent a molecular mechanism describing the transient nature of seizures experienced by patients with disease-associated null *GRIN2A* variants. Unfortunately, current technical limits such as *Grin2a*^−/−^ mice not experiencing spontaneous seizures regardless of age and the lack of a reliable seizure-inducing paradigm for neonatal mice represent an important caveat and preclude elucidation of a molecular mechanism for the transient seizure susceptibility of disease-associated null *GRIN2A* patients. Many questions remain unexplored, including how this transient delay is overcome purely with aging and how this delay in functional maturation may impact synaptic plasticity and neural oscillations. It seems likely that other aspects of circuit function may still be perturbed despite PV cells from *Grin2a*^*+/−*^ and *Grin2a*^*−/−*^ mice attaining *Grin2a*^*+/+*^ electrophysiological function in a delayed fashion. Nevertheless, the delay in PV cell maturation will diminish capacity of inhibition to prevent high frequency firing, which is broadly accepted as one condition promoting epileptiform activity.

The data we report here suggest multiple potential roles for the GluN2A subunit of NMDARs in the regulation of function, development, and maturation of GABAergic interneurons in the mouse hippocampus. Specifically, we report that full loss of *Grin2a* causes aberrations in PV cell density. We show at P6-8, the evoked NMDAR-mediated charge transfer between *Grin2a*^+/+^ mice and *Grin2a*^+/−^ mice are identical, while *Grin2a*^−/−^ mice allow much more charge (and calcium) into the neuron per synaptic event involving NMDARs. These data may explain why *Grin2a*^−/−^ mice display an increased number of PV cells, while the number of PV cells between *Grin2a*^+/+^ and *Grin2a*^+/−^ mice are unaltered. Our data also supports findings that increased pyramidal cell activity during neonatal development can modulate MGE-derived interneuron density^50^. Despite this similarity, Wong et al. report that increased pyramidal cell activity promotes a decrease in PV cell density^50^. While the exact mechanisms controlling interneuron survival are still not elucidated, our study highlights that perhaps interneuron survival rules differ between the neocortex and hippocampus^50^. Alternatively, it is plausible that increased circuit activity from conception as in our study may promote differential interneuron survival cues than those induced by increasing activity in just pyramidal cells at P5^50^.

Our data suggest that the loss of GluN2A may be impacting interneuron survival or death which are controlled by a combination of internal genetic cues, external neurotropic factors, and early interneuron electrical activity^57–59^. The extent to which local electrical signaling, especially from NMDARs, within PV interneurons controls pro-death pathways has not been determined. The loss of *Grin2a* only impacts PV cell density, but not CCK cell density, which may suggest that MGE-derived interneurons are preferentially affected. The lack of effect on CCK cell density in *Grin2a*^−/−^ mice could be due to CCK cells having little functional GluN2A expression^29,44,51^. In addition to changes in PV cell density, we also report that the total loss of *Grin2a* generates an upregulation of *Grin2d* and *Grin3a* mRNA. Given that PV cells highly express GluN2D-containing NMDARs^32,60^, these two findings may coincide. However, there is little evidence of *Grin3a* expression on PV cells^61,62^. Instead, recent studies highlight immense GluN3A-mediated NMDA currents on somatostatin interneurons^62^, which are also generated from the MGE interneuron stem cell population. Thus, PV cells may not be the only interneuron subtype that is dysfunctional in *Grin2a*^*−/−*^ mice. Future experiments should focus on elucidating electrophysiological function of other MGE-derived interneurons, such as somatostatin cells and some neurogliaform cells.

We also show that CA1 PV cells from *Grin2a*^+/+^ mice display age-dependent alterations in passive electrical and action potential firing properties. By examining both passive intrinsic properties and action potential firing characteristics, we can estimate how these cells may respond to excitatory afferent activity and how efficiently they can transduce somatic depolarizations into signal-carrying action potentials. In both *Grin2a*^*+/−*^ and *Grin2a*^*−/−*^ mice we show age- and *Grin2a* gene dosing-dependent transient prolongations of the membrane time constant, increases in input resistance, and longer action potential half-widths. Each of these electrophysiological measures are known to be age-dependent, with PV cells in neonatal mice displaying a higher input resistance, longer membrane time constant, and longer action potential half-widths than PV cells from preadolescent mice^40,53–55^. Moreover, PV cells from *Grin2a*^*+/−*^ and *Grin2a*^−/−^ mice have a lower threshold to reach depolarization-induced block of action potential firing and lower peak action potential firing frequencies. These latter electrophysiological characteristics could lead to failure of circuit level inhibition as interneuron depolarization increases, consistent with a hyperexcitable phenotype.

The transcriptional shift from immature to mature PV electrophysiological properties has been shown to be activity-dependent^63,64^, and the loss of GluN2A-mediated signaling appears to slow cellular maturation of CA1 PV cells. However, it is currently unknown whether these delays stem from a loss of GluN2A-mediated signaling on PV cell dendrites (cell autonomous) or due to excitability changes of local pyramidal cells (non-cell autonomous). It is also unknown how the loss of GluN2A-mediated signaling impacts the age-dependent maturation of perineuronal nets, which previous data has suggested may be altered in *Grin2a*^−/−^ mice^65^. We show that all measured electrophysiological properties ultimately reach *Grin2a*^*+/+*^ levels in adult mice, suggesting that although GluN2A signaling may be sufficient to initiate these maturation transcriptional programs, it is not required. While all ramifications of a prolonged immature electrophysiological profile in developing PV cells are not known, this delay in PV cell maturation will diminish the strength of inhibition during a period that coincides with critical period plasticity in various brain regions^66,67^. Thus, this delay could critically alter circuit formation in the hippocampus and other regions. Larger input resistances and lower peak action potential firing frequencies will dampen the temporal resolution of inhibitory tone in a developing network, which may promote maladaptive plasticity and incorrect circuit wiring. In addition, changes in depolarization-induced block of action potentials correlates with neural excitability and propagation of epileptiform activity^68^. Thus, disruption of PV cell function during development will likely have profound consequences for eventual mature networks that arise. Our findings showcase profound dysfunction in a major class of somatic-targeting GABAergic interneurons during a critical period of neurodevelopment. It is unequivocal that dysfunctional PV cells during preadolescent development will be devasting to circuit function and could be the catalyst for generating a hyperexcitable network. Future studies should be aimed at uncovering how aberrant PV cells attain wild-type function with age and whether these compensatory mechanisms can be attributed to the transient seizure activity we describe in disease-associated null *GRIN2A* patients.

### Supplementary information

Supplementary Information

Description of Additional Supplementary Files

Supplemental Data 1

Supplemental Data 2

Supplemental Data 3

Reporting Summary

## Data Availability

All data are available from the corresponding author on reasonable request. No custom computer code was used in the generation of data presented. Source data for all main figures can be found in Supplemental Data 1 (Figs. 1–2), Supplemental Data 2 (Figs. 3–4), and Supplemental Data 3 (Figs. 5–8).
