# Supplementary material for: Loss of Grin2a causes a transient delay in the electrophysiological maturation of hippocampal parvalbumin interneurons
Source: Commun Biol. 2023 Sep 19;6:952. doi: 10.1038/s42003-023-05298-9 (PMC10507040; doi:10.1038/s42003-023-05298-9)
Supplement: Supplementary file 1 — Supplementary Information [file 42003_2023_5298_MOESM1_ESM.pdf]

Loss of *Grin2a* Causes a Transient Delay in the Electrophysiological Maturation of Hippocampal Parvalbumin Interneurons

Chad R. Camp[1], Anna Vlachos[2], Chiara Klöckner[3], Ilona Krey[3], Tue G. Banke[1], Nima Shariatzadeh[1], Sarah M .Ruggiero[4,5], Peter Galer[6], Kristen L. Park[7], Adam Caccavano[2], Sarah Kimmel[2], Xiaoqing Yuan[2], Hongjie Yuan[1,8], Ingo Helbig[4,5,6,9], Tim A. Benke[7], Johannes R. Lemke[3,10], Kenneth A. Pelkey[2], Chris J. McBain[2], Stephen F. Traynelis[1,8,11]

[1]Department of Pharmacology and Chemical Biology, Emory University School of Medicine, Atlanta, GA 30322
[2]Section on Cellular and Synaptic Physiology, Eunice Kennedy-Shriver National Institute of Child Health and Human Development, National Institutes of Health, Bethesda, MD 20892.
[3]Institute of Human Genetics, University of Leipzig Medical Center, Leipzig Germany
[4]Division of Neurology, Children's Hospital of Philadelphia, Philadelphia, PA 19104
[5]The Epilepsy NeuroGenetics Initiative, Children's Hospital of Philadelphia, Philadelphia, PA 19104, USA
[6]Department of Biomedical and Health Informatics, Children's Hospital of Philadelphia, Philadelphia, PA 19146, USA
[7]University of Colorado School of Medicine and Children's Hospital Colorado, Aurora CO, 80045
[8]Center for Functional Evaluation of Rare Variants, Emory University School of Medicine, Atlanta, GA 30322
[9]Department of Neurology, University of Pennsylvania Perelman School of Medicine, Philadelphia, PA 19104, USA
[10]Center for Rare Diseases, University of Leipzig Medical Center, Leipzig Germany
[11]Center for Neurodegenerative Disease, Emory University School of Medicine, Atlanta, GA 30322
Corresponding author email: strayne@emory.edu

Supplementary File

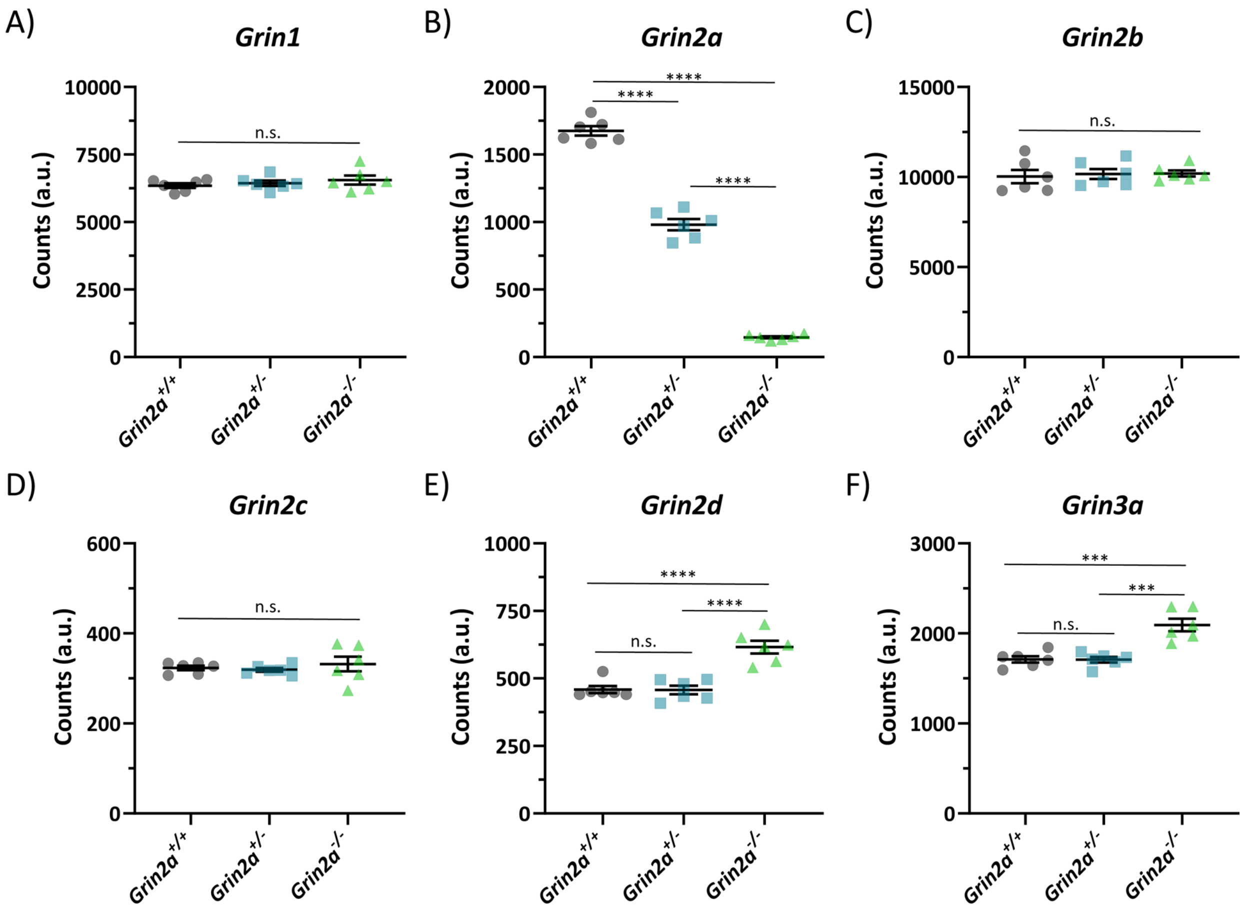

Supplemental Figure S1. Evaluation of all *GRIN* gene transcripts in developing hippocampus. Whole hippocampi from juvenile mice across 18 samples, six from each genotype, were subjected to NanoString gene expression analysis. Normalized raw counts were then plotted and tested for statistical significance, controlling for false-discovery rate. There were no significant differences in **A)** *Grin1*, **C)** *Grin2b*, and **D)** *Grin2c* gene expression across all three genotypes. As expected, **B)** *Grin2a* gene expression shows a statistically significant, genotype-dependent decrease. Both **E)** *Grin2d* and **F)** *Grin3a* genes were statistically significantly upregulated in *Grin2a*$^{-/-}$ mice compared to *Grin2a*$^{+/+}$, with no significant differences between *Grin2a*$^{+/+}$ and *Grin2a*$^{+/-}$ mice. Counts for the *Grin3b* gene fell below our limit of detection as determined by negative controls. All statistical tests performed were one-way ANOVAs, with multiple post-hoc comparisons. *** = p<0.001; **** = p<0.0001; n.s. = not significant; a.u. = arbitrary units.

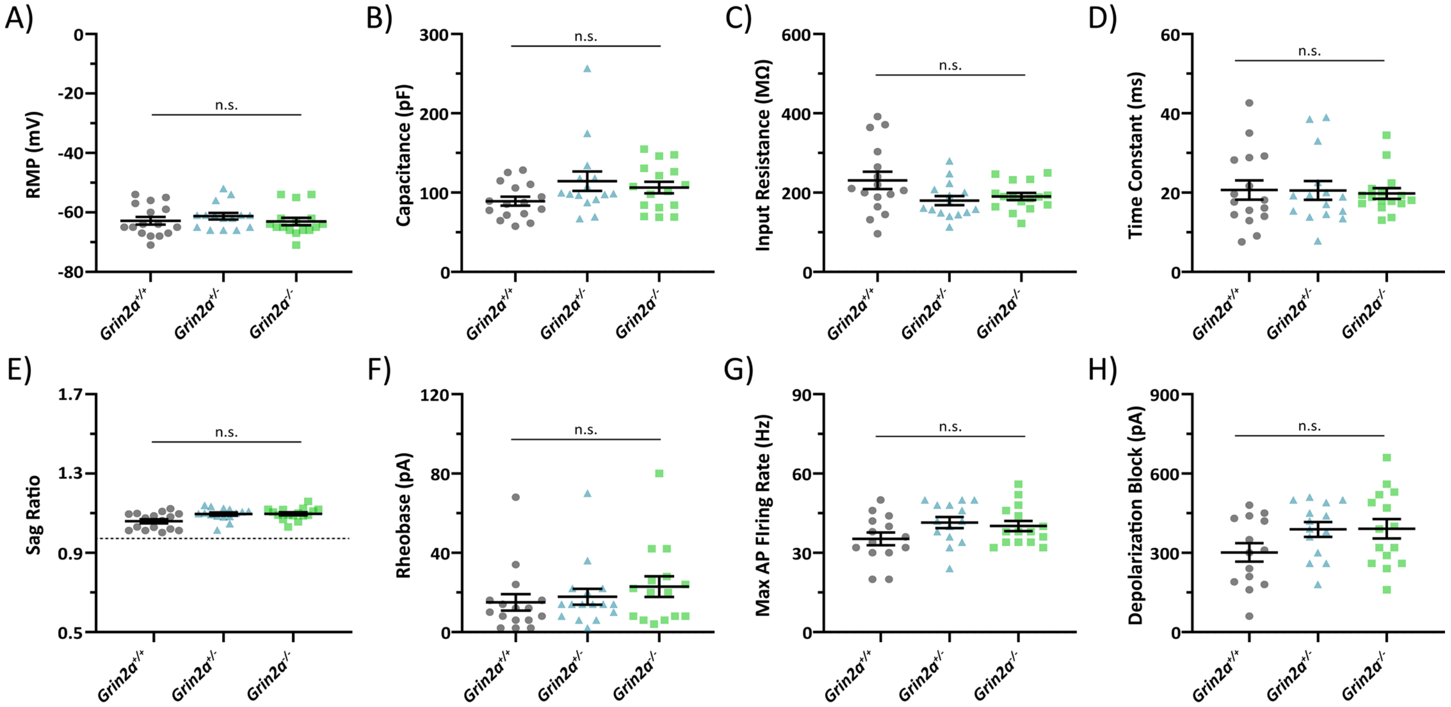

Supplemental Figure S2. Juvenile CA1 circuit hyperexcitability is not due to changes in CA1 pyramidal cell intrinsic or action potential firing properties. Data from juvenile (P14-16) CA1 pyramidal cells show no significant differences in **A)** resting membrane potential, **B)** cell capacitance, **C)** input resistance, **D)** membrane time constant, **E)** sag ratio, **F)** rheobase, **G)** maximum action potential firing rate, or **H)** current required to reach depolarization-induced blockade of action potential firing when assayed via one-way ANOVA. Data represented show mean ± SEM. RMP = resting membrane potential; AP = action potential; depolarization block = current required to reach depolarization-induced blockade of action potential firing; n.s. = not significant.

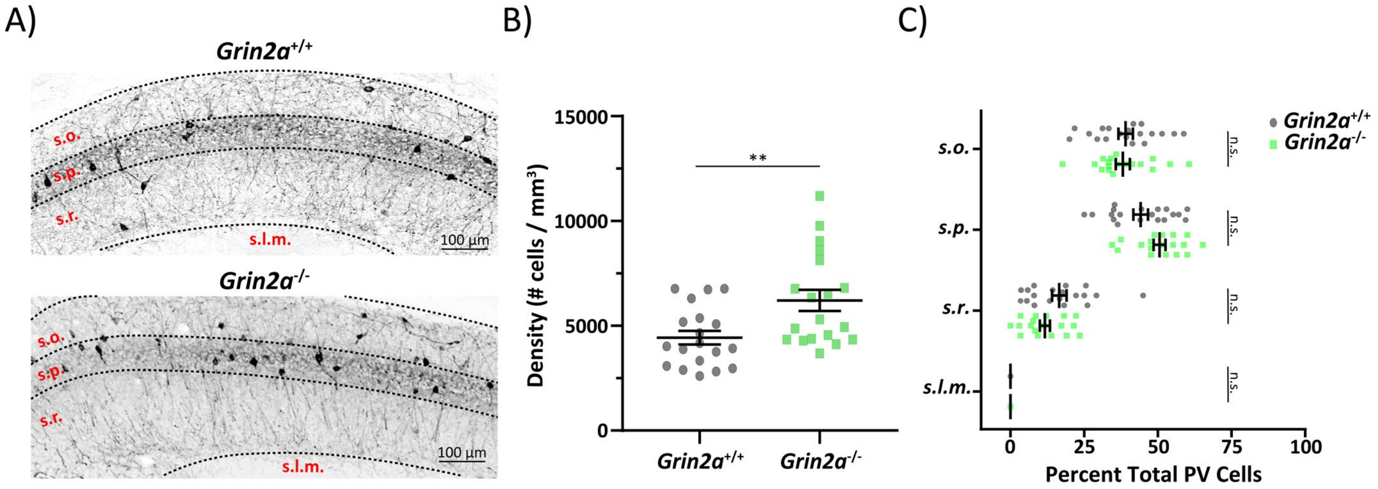

Supplemental Figure S3. CA1 PV cell density remains increased in adult *Grin2a*$^{-/-}$ mice. **A)** Representative images of CA1 hippocampal sections stained for PV in adult mice. **B)** CA1 PV cell density in adult *Grin2a*$^{-/-}$ mice remains increased compared to *Grin2a*$^{+/+}$ (unpaired Student's t-test, p=0.0055). **C)** There is no difference in PV CA1 cellular lamination in adult *Grin2a*$^{-/-}$ and *Grin2a*$^{+/+}$ mice. Data represented show mean ± SEM. *s.o. = stratum oriens*; *s.p. = stratum pyramidale*; *s.r. = stratum radiatum*; *s.l.m. = stratum lacunosum moleculare*; PV = parvalbumin; ** = p < 0.01; n.s. = not significant.

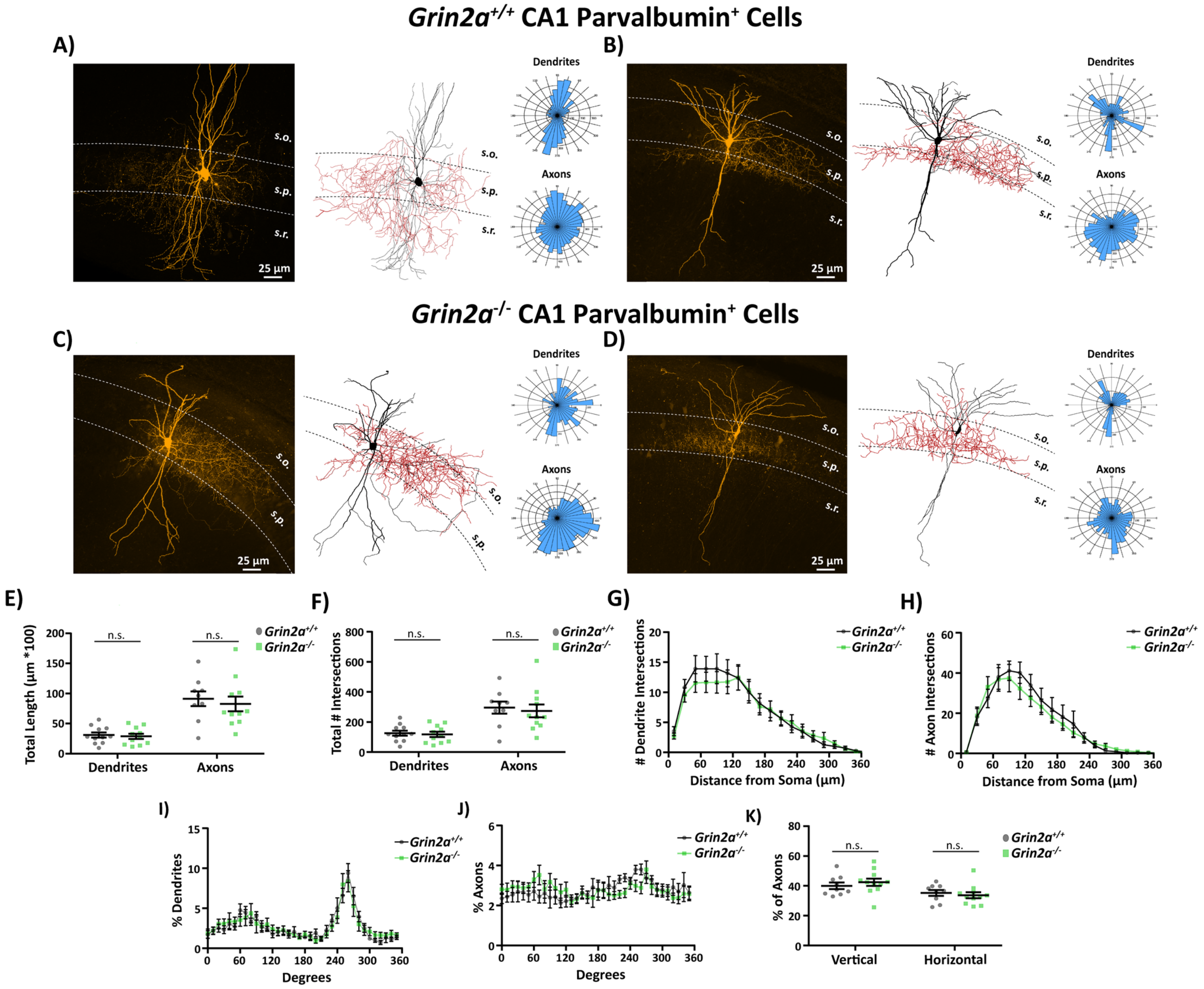

Supplemental Figure S4. There is no difference in CA1 PV cell morphology in preadolescent *Grin2a*$^{-/-}$ mice. Representative images for **A-B)** *Grin2a*$^{+/+}$ or **C-D)** *Grin2a*$^{-/-}$ biocytin backfilled CA1 PV cells shown as maximum intensity projection images with accompanying NeuroLucida tracings (black for dendrites and red for axons) and polar plots for both dendrites and axons. There is no change in **E)** total length of dendrites or axons or in **F)** the total number of Sholl intersections in dendrites or axons in preadolescent CA1 PV cells from either genotype. Sholl analysis of both **G)** dendrites and **H)** axons from preadolescent CA1 PV cells are also unchanged across genotype. The placement of both **I)** dendrites or **J)** axons assessed via polar plots showed no differences, with **K)** equal axonal placement in both the vertical and horizontal planes. Data obtained from 9-11 slices across 4 animals per genotype. Symbols are mean ± SEM. n.s. = not significant. s.o. = *stratum oriens*; s.p. = *stratum pyramidale*; s.r. = *stratum radiatum*

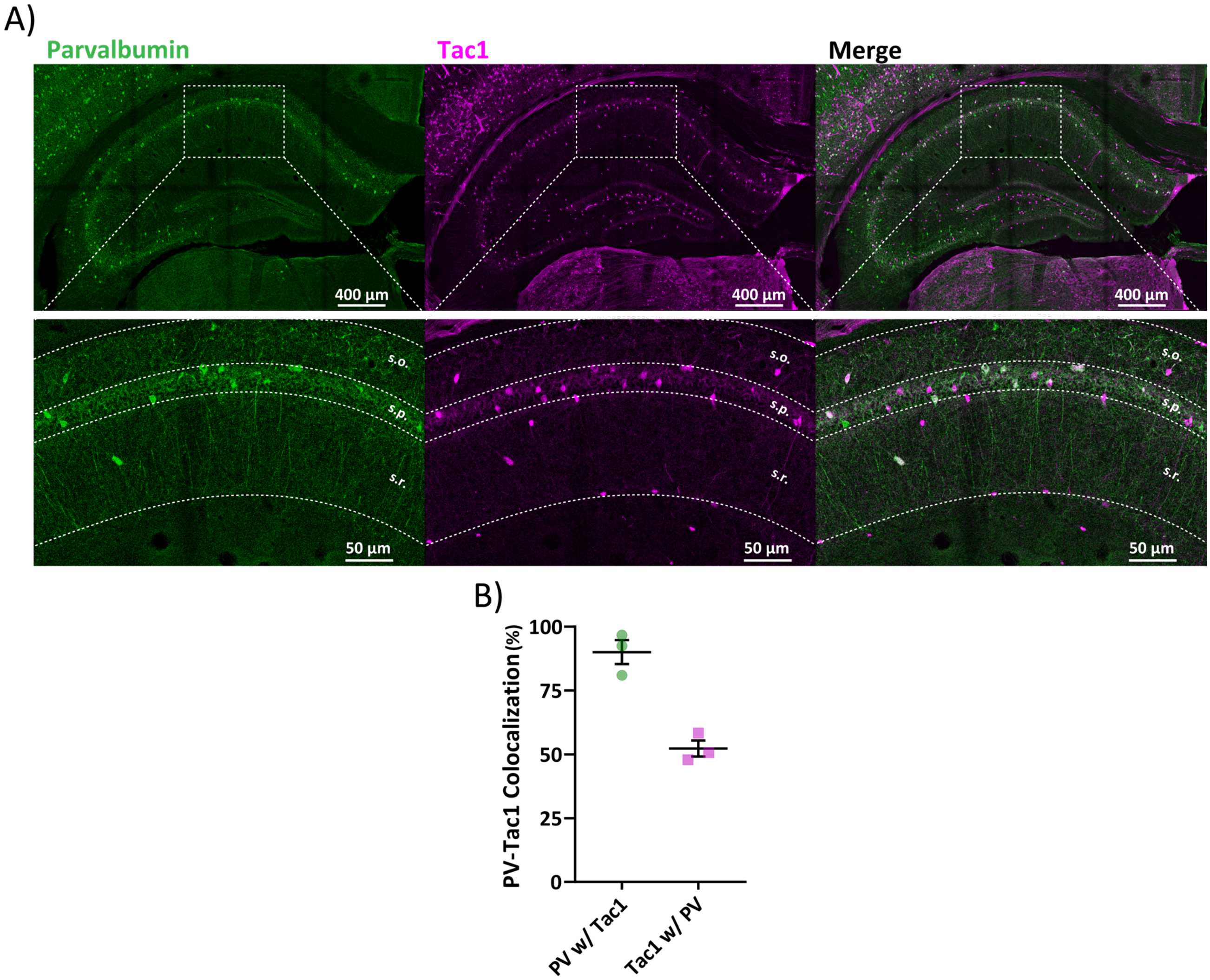

Supplemental Figure S5. The majority of cells that are immunopositive for parvalbumin are also immunopositive for Tac1. **A)** Example hippocampal slice from an adult *Tac1*-Cre x Floxed-eGFP mouse that has been stained for anti-parvalbumin and anti-eGFP. Inset displays a more detailed view of CA1, with the majority of PV-positive cells in *stratum oriens* and *stratum pyramidale* also being immunopositive for Tac1. **B)** Quantification of PV and Tac1 co-expression overlap. Since CA1 *stratum oriens* and *stratum pyramidale* had the most extensive overlap for PV and Tac1, all recordings made from *Tac1*-positive cells were chosen in these two layers. *s.o.* = *stratum oriens*; *s.p.* = *stratum pyramidale*; *s.r.* = *stratum radiatum*.

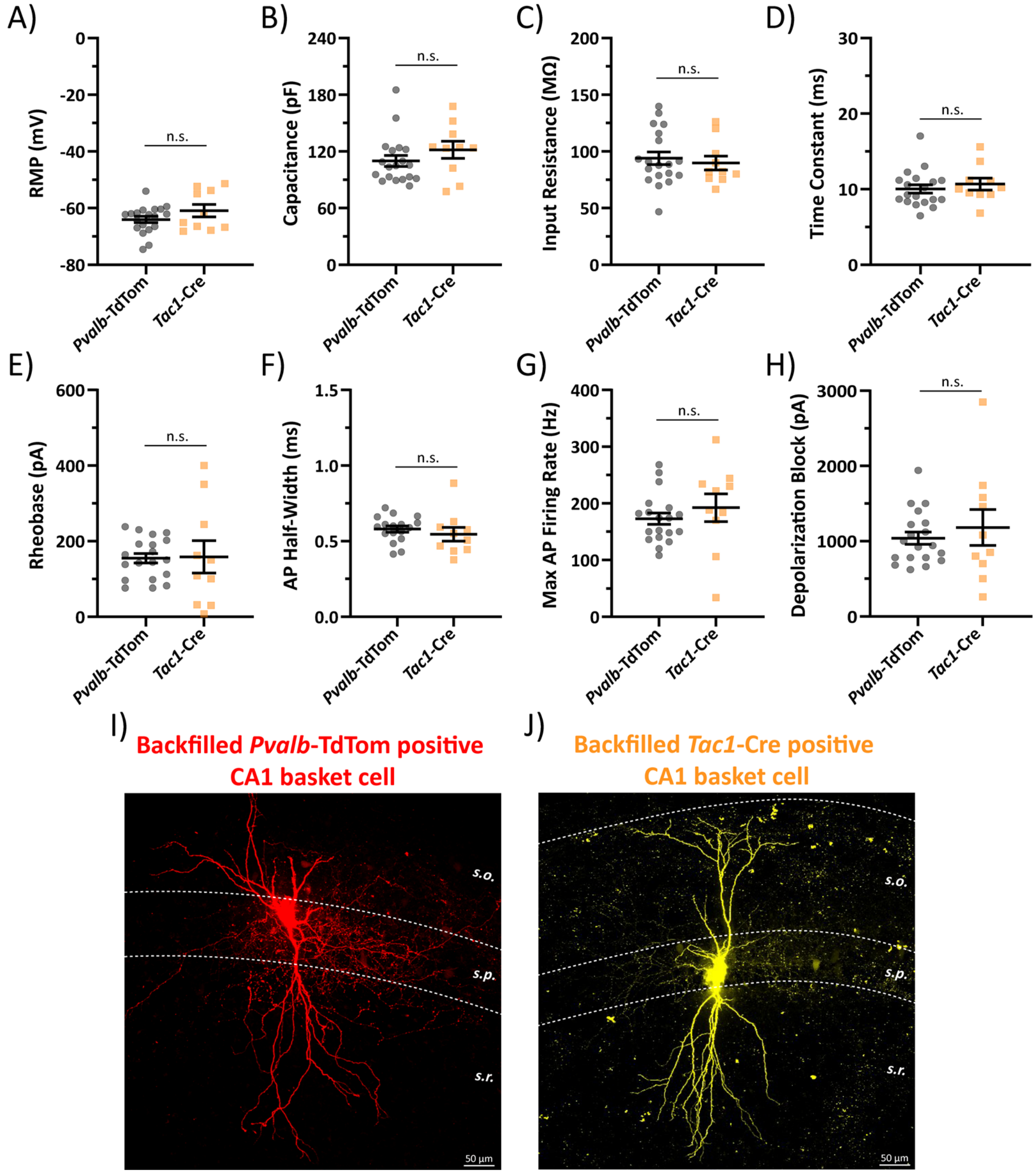

Supplemental Figure S6. *Pvalb*-TdTom identified CA1 PV interneurons display identical electrophysiological properties as CA1 PV interneurons identified via *Tac1*-Cre. **A-H)** Both passive and action potential firing electrophysiological properties of juvenile CA1 PV cells from *Pvalb*-TdTom and *Tac1*-Cre show no statistically significant differences. Example backfills of CA1 PV cells using **I)** *Pvalb*-TdTom and **J)** *Tac1*-Cre mouse lines indicates that both can be reliably used to visualize PV basket cells in CA1 *stratum pyramidale*. RMP = resting membrane potential; AP = action potential; n.s. = not significant; *s.o. = stratum oriens*; *s.p. = stratum pyramidale*; *s.r. = stratum radiatum*.

Supplemental Table S1. Null *GRIN2A* variant patient data with time when seizures began and/or ended. Data represented are gathered from the *GRIN* portal (https://grin-portal.broadinstitute.org/), or respective patient registries housed at University of Leipzig, Children's Hospital of Philadelphia, or University of Colorado School of Medicine. Seizure offset is defined as freedom from seizures for two consecutive years. N/A = not applicable. '--' = data not available at time of publication. Some patients had not reached an age where offset was expected.

Gene	Variant Type	Nucleotide Information	Protein Information	Amino Acid Number	Seizure Onset (Years)	Seizure Offset (Years)
GRIN2A	Deletion	N/A	del exon 1-3	N/A	10	--
GRIN2A	Deletion	chr16:10,227,326-10,300,839x1	del exon 1-3	N/A	7	--
GRIN2A	Deletion	chr16:10,227,326-10,300,839x1	del exon 1-3	N/A	3	--
GRIN2A	Deletion	chr16:10,227,326-10,300,839x1	del exon 1-3	N/A	10	--
GRIN2A	Deletion	chr16:10,227,326-10,300,839x1	del exon 1-3	N/A	2.5	--
GRIN2A	Deletion	chr16:10,241,998-10,300,800x1	del exon 1-3	N/A	8	--
GRIN2A	Deletion	chr16:10,241,998-10,300,800x1	del exon 1-3	N/A	6	--
GRIN2A	Deletion	chr16:10,246,239<U+0096>10,321,593<d7>1	del exon 1-3	N/A	6	--
GRIN2A	Deletion	chr16:10,246,239<U+0096>10,321,593<d7>1	del exon 1-3	N/A	6	--
GRIN2A	Deletion	chr16:10,246,239-10,354,862x1	del exon 1-3	N/A	4	--
GRIN2A	Deletion	possible c.1-?	Deletion exon 3	possible 1-?	6	6
GRIN2A	Deletion	--	Deletion exon 3	--	6.6	--
GRIN2A	Deletion	c.280_283delCGCA	p.(Arg94Serfs*15)	94	6.2	12.1
GRIN2A	Deletion	c.280_283delCGCA	p.(Arg94Serfs*15)	94	6.2	12.2
GRIN2A	Deletion	possible 415; chr16:10,000,670-10,197,654x1	del exon 4	possible 139-?	2	--
GRIN2A	Deletion	16p13 deletion	del exon-?	possible 139-?	2	--
GRIN2A	Deletion	16p13.2 292,09 kb	del exon 4-14	possible 139-?	2	--
GRIN2A	Deletion	16p13.2 deletion (6907020-10415739)	--	--	2.4	--
GRIN2A	Deletion	c.(414+1_415-1)_(1007+1_1008-1)	del exon 4	possible 139-?	4	--
GRIN2A	Deletion	possible c.415; chr16:9,825,755-10,069,792x1	del exon 4-14	possible 139-?	7.7	--
GRIN2A	Deletion	possible c.415-?; del 292,09kb	del exon 4-14	possible 139-?	4	--
GRIN2A	Deletion	possible c.415-?	Deletion exon 4	possible 139-?	7	12
GRIN2A	Deletion	possible c.415-?	Deletion exon 4	possible 139-?	5	5
GRIN2A	Deletion	possible c.415-?	del exon 4	possible 139-?	3	--
GRIN2A	Deletion	possible c.415-1122	dup exon 4-5	possible 139-?	0.25	--
GRIN2A	Deletion	possible c.1009-?	Deletion exon 5	possible 337-?	1.5	--
GRIN2A	Deletion	possible c.1123-?	Deletion exon 6	possible 375-?	3	--
GRIN2A	Deletion	possible c.1123-?	del exon 6-11	possible 375-?	3	--
GRIN2A	Deletion	possible c.1123-?	del exon 6-7	possible 375-?	3	--
GRIN2A	Deletion	possible c.1123-?	del exon 6-7	possible 375-?	1	--
GRIN2A	Deletion	possible c.1123-?	del exon 6-7	possible 375-?	8	--
GRIN2A	Deletion	c.1585delG	p.(Val529Trpfs*22)	529	2	--
GRIN2A	Deletion	c.1586delT	p.(Val529Glyfs*22)	529	5.75	--
GRIN2A	Deletion	c.1650_1651delAGGTGTGT	p.(Leu-?)	550	4	8
GRIN2A	Deletion	c.1650_1651delAGGTGTGT	--	--	--	11
GRIN2A	Deletion	c.1651+1del	p.(Glu-?)	551	7	--
GRIN2A	Deletion	c.1686del	p.(Phe562Leufs*2)	562	2	--
GRIN2A	Deletion	chr16:9,915,756<U+0096>9,915,815<d7>1	del exon 11	possible 670-?	5	--
GRIN2A	Deletion	chr16:9,915,756<U+0096>9,915,815<d7>1	del exon 11	possible 670-?	5	--
GRIN2A	Deletion	chr16:9,915,756<U+0096>9,915,815<d7>1	del exon 11	possible 670-?	4.5	--
GRIN2A	Deletion	possible c.2170-?	del exon 12-14	possible 724-?	--	12
GRIN2A	Deletion	c.2334_2338delCTTGC	p.(Leu779Serfs*5)	779	4	--
GRIN2A	Deletion	c.2408del	p.(Glu803Glyfs*5)	803	2	--
GRIN2A	Deletion	c.3596delC	p.(Pro1199Argfs*32)	1199	--	10.2
GRIN2A	Deletion	c.3596delC	p.(Pro1199Argfs*32)	1199	--	10.2
GRIN2A	Del/Insert	c.2341_2343delinsAT	p.(Gln781Ilefs*27)	781	6	--
GRIN2A	Duplication	c.90dupT	p.(Pro31Serfs*107)	31	4	--
GRIN2A	Duplication	c.176_179dupAGGC	p.(Ala61Glyfs*78)	61	6	--
GRIN2A	Duplication	possible c.415-1122	dup exon 4-5	possible 139-?	0.25	--
GRIN2A	Duplication	c.2007+2dup	p.(Lys-?)	669	10	--
GRIN2A	Duplication	c.2253dupG	p.(Ser752Glufs*34)	752	5	--
GRIN2A	Nonsense	c.2T>C	p.(Met1?)	1	3.5	--
GRIN2A	Nonsense	c.2T>C	p.(Met1?)	1	2	--

GRIN2A	Nonsense	?	p.(Ala27Glyfs*112)	27	--	16
GRIN2A	Nonsense	c.172G>T	p.(Glu58*)	58	5	--
GRIN2A	Nonsense	?	p.(Ala61Glyfs*78)	61	--	8
GRIN2A	Nonsense	c.500G>A	p.(Trp167*)	167	4	--
GRIN2A	Nonsense	c.594G>A	p.(Trp198*)	198	5	--
GRIN2A	Nonsense	c.594G>A	p.(Trp198*)	198	4	--
GRIN2A	Nonsense	c.594G>A	p.(Trp198*)	198	3	--
GRIN2A	Nonsense	?	p.(Trp198*)	198	--	12
GRIN2A	Frame shift	c.627delC	p.Phe210Leufsx10	210	7.75	15.1
GRIN2A	Nonsense	c.652C>T	p.(Gln218*)	218	1	--
GRIN2A	Nonsense	c.652C>T	p.(Gln218*)	218	0.9	--
GRIN2A	Nonsense	c.703G>T	p.Glu235X	235	3	9
GRIN2A	Nonsense	c.1001T>A	p.(Leu334*)	334	3	--
GRIN2A	Nonsense	c.1001T>A	p.(Leu334*)	334	1	--
GRIN2A	Nonsense	c.1001T>A	p.(Leu334*)	334	4	--
GRIN2A	Nonsense	c.1036A>T	p.Lys346Ter	346	11	--
GRIN2A	Nonsense	C.1115G>A	p.Trp372	372	0.04	3
GRIN2A	Nonsense	?	p.(Pro415Hisfs*8)	415	--	10
GRIN2A	Nonsense	?	p.(Pro415Hisfs*8)	415	--	20
GRIN2A	Nonsense	c.1613C>G	p.(Ser538*)	538	4	--
GRIN2A	Nonsense	c.1818G>A	p.(Trp606*)	606	3.5	--
GRIN2A	Nonsense	c.2041C>T	p.(Arg681*)	681	4	--
GRIN2A	Nonsense	c.2041C>T	p.(Arg681*)	681	2.5	--
GRIN2A	Nonsense	c.2407G>T	p.(Glu803*)	803	3.5	--
GRIN2A	Nonsense	?	p.(Arg847*)	847	--	10.8
GRIN2A	Unknown	c.415-2A>G	p.(Asp-?)	139	10	--
GRIN2A	Unknown	c.1007+1G>A	p.(?)	336	6	--
GRIN2A	Unknown	c.1007+1G>A	p.(?)	336	3	--
GRIN2A	Unknown	c.1007+1G>A	p.(?)	336	4	--
GRIN2A	Unknown	c.1007+1G>A	p.(?)	336	6	--
GRIN2A	Unknown	c.1007+1G>A	p.(?)	336	1.5	--
GRIN2A	Unknown	c.1007+1G>A	p.(?)	336	2	--
GRIN2A	Unknown	c.1007+1G>A	p.(?)	336	4	--
GRIN2A	Unknown	c.1007+1G>A	p.(?)	336	4	--
GRIN2A	Unknown	c.1007+1G>A	p.(?)	336	5.5	--
GRIN2A	Unknown	c.1007+1G>A	p.(?)	336	2.5	--
GRIN2A	Unknown	c.1007+1G>A	p.(?)	336	6	--
GRIN2A	Unknown	c.1007+1G>A	p.(?)	336	10	--
GRIN2A	Unknown	c.1007+1G>A	p.(?)	336	--	9
GRIN2A	Unknown	c.1007+1G>T	p.(?)	336	4	--
GRIN2A	Unknown	c.1123-1G>T	p.(Val-?)	375	4	--
GRIN2A	Unknown	c.1123-1G>T	p.(Val-?)	375	3.5	--
GRIN2A	Unknown	c.1123-2A>G	p.(Val-?)	375	2	--
GRIN2A	Unknown	c.1123-2A>G	p.(Val-?)	375	4	--
GRIN2A	Unknown	c.1123-2A>G	p.(Val-?)	375	6	--
GRIN2A	Unknown	c.1123-2A>G	p.(Val-?)	375	5	--
GRIN2A	Unknown	c.1123-2A>G	p.(Val-?)	375	4	--
GRIN2A	Unknown	c.1123-2A>G	p.(Val-?)	375	5	--
GRIN2A	Unknown	c.1123-2A>G	p.(Val-?)	375	5	--
GRIN2A	Unknown	c.2007+1G>A	p.(Lys-?)	669	3.5	--

Supplemental Table S2. Missense *GRIN2A* variant patient data with information on when seizure susceptibility began and/or ended. Data represented are gathered from the *GRIN* portal (https://grin-portal.broadinstitute.org/), University of Leipzig, Children's Hospital of Philadelphia, or University of Colorado Anshutz Medical Center. Seizure offset is defined as freedom from seizures for two consecutive years. '--' = data not available at time of publication.

Gene	Variant Type	Nucleotide Information	Protein Information	Amino Acid Number	Seizure Onset (Years)	Seizure Offset (Years)
GRIN2A	Missense	c.1667C>T	p.(Arg217Trp)	217	0.25	1.7
GRIN2A	Missense	c.692G>A	p.(Cys231Tyr)	231	3	--
GRIN2A	Missense	c.1232T>A	p.(Leu411Gln)	411	3	--
GRIN2A	Missense	c.1251C>T	p.(Val417Val)	417	0.8	--
GRIN2A	Missense	c.1306T>C	p.(Cys436Arg)	436	4	--
GRIN2A	Missense	c.1447G>A	p.(Gly483Arg)	483	4	--
GRIN2A	Missense	c.1492G>A	p.(Gly498Ser)	498	8	--
GRIN2A	Missense	c.1532C>T	p.(Ser511Leu)	511	2.5	--
GRIN2A	Missense	c.1552C>T	p.(Arg518Cys)	518	3	--
GRIN2A	Missense	c.1553G>A	p.(Arg518His)	518	3	--
GRIN2A	Missense	c.1553G>A	p.(Arg518His)	518	2	--
GRIN2A	Missense	c.1592C>T	p.(Thr531Met)	531	6.7	--
GRIN2A	Missense	c.1592C>T	p.(Thr531Met)	531	7	--
GRIN2A	Missense	c.1592C>T	p.(Thr531Met)	531	11	--
GRIN2A	Missense	c.1642G>A	p.(Ala548Thr)	548	6	--
GRIN2A	Missense	c.1655C>G	p.(Pro552Arg)	552	0.75	--
GRIN2A	Missense	c.1655C>G	p.(Pro552Arg)	552	0.75	--
GRIN2A	Missense	c.1841A>G	p.(Asn614Ser)	614	0.4	--
GRIN2A	Missense	c.1845C>A	p.(Asn615Lys)	615	0.75	--
GRIN2A	Missense	c.1845C>A	p.(Asn615Lys)	615	0.25	--
GRIN2A	Missense	c.1903G>A	p.(Ala635Thr)	635	0.6	--
GRIN2A	Missense	c.1930A>G	p.(Ser644Gly)	644	0.5	--
GRIN2A	Missense	c.1936A>G	p.(Thr646Ala)	646	0.7	--
GRIN2A	Missense	c.1936A>G	p.(Thr646Ala)	646	0.25	--
GRIN2A	Missense	c.1945C>G	p.(Leu649Val)	649	0.75	--
GRIN2A	Missense	c.1946_1947delin	p.(Leu649Pro)	649	1	--
GRIN2A	Missense	c.1948G>T	p.(Ala650Ser)	650	1.5	--
GRIN2A	Missense	c.1954T>G	p.(Phe652Val)	652	2	--
GRIN2A	Missense	c.1961T>C	p.(Ile654Thr)	654	0.2	--
GRIN2A	Missense	c.2081T>C	p.(Ile694Thr)	694	2.5	--
GRIN2A	Missense	c.2095C>T	p.(Pro699Ser)	699	8	--
GRIN2A	Missense	c.2113A>G	p.(Met705Val)	705	8	--
GRIN2A	Missense	c.2191 G>A	p.Asp731Asn	731	1.9	--
GRIN2A	Missense	c.2191G>A	p.(Asp731Asn)	731	4	--
GRIN2A	Missense	c.2191G>A	p.Asp731Asn	731	3.9	--
GRIN2A	Missense	c.2326G>T	p.(Asp776Tyr)	776	7	--
GRIN2A	Missense	c.2434C>A	p.(Leu812Met)	812	1.5	--
GRIN2A	Missense	c.2449A>G	p.(Met817Val)	817	2	--
GRIN2A	Missense	c.2450T>C	p.(Met817Thr)	817	2	--
GRIN2A	Missense	c.2450T>C	p.(Met817Thr)	817	1.25	--
GRIN2A	Missense	c.2452G>A	p.(Ala818Thr)	818	3	--
GRIN2A	Missense	c.2453C>A	p.(Ala818Glu)	818	1	--
GRIN2A	Missense	c.2907C>G	p.(Asn969Lys)	969	2.5	--
GRIN2A	Missense	c.3664C>G	p.(Pro1222Ala)	1222	7.9	--
GRIN2A	Missense	c.3664C>G	p.(Pro1222Ala)	1222	7.8	--

Supplemental Table S3. Quality control metrics from NanoString transcriptomic screen of NMDAR subunit genes. Imaging QC is the ratio of the observed counts per field of view divided by the total counts (n=555) in each field of view. Binding density is the number of barcodes read per µm^2. QC = quality controls.

	RNA Integrity Number (RIN)	Imaging QC	Binding Density QC	Limit of Detection	Positive Control Linearity
***Grin2a*$^{+/+}$ Mouse #1**	9.8	0.98	0.28	43.8	1
***Grin2a*$^{+/+}$ Mouse #2**	9.8	0.98	0.26	39.7	1
***Grin2a*$^{+/+}$ Mouse #3**	9.9	0.98	0.27	41.1	1
***Grin2a*$^{+/+}$ Mouse #4**	9.8	0.98	0.27	42.9	1
***Grin2a*$^{+/+}$ Mouse #5**	9.9	0.94	0.3	38.2	1
***Grin2a*$^{+/+}$ Mouse #6**	8.8	0.91	0.31	44.2	1
***Grin2a*$^{+/+}$ Mouse #1**	8.4	0.98	0.27	34.5	1
***Grin2a*$^{+/-}$ Mouse #2**	8.6	0.99	0.25	30	1
***Grin2a*$^{+/-}$ Mouse #3**	8.4	0.98	0.25	41.6	1
***Grin2a*$^{+/-}$ Mouse #4**	9	0.97	0.28	38	1
***Grin2a*$^{+/-}$ Mouse #5**	9.2	0.92	0.27	32.5	1
***Grin2a*$^{+/-}$ Mouse #6**	7.5	0.98	0.27	41.2	0.99
***Grin2a*$^{-/-}$ Mouse #1**	7.7	0.97	0.34	43.3	1
***Grin2a*$^{-/-}$ Mouse #2**	7.9	0.97	0.32	38.4	1
***Grin2a*$^{-/-}$ Mouse #3**	7.7	0.97	0.33	37.7	1
***Grin2a*$^{-/-}$ Mouse #4**	9.4	0.97	0.33	35.8	0.99
***Grin2a*$^{-/-}$ Mouse #5**	9.4	0.97	0.33	49.4	1
***Grin2a*$^{-/-}$ Mouse #6**	9.7	0.89	0.4	43.2	1

Supplemental Table S4. Action potential spiking probability in *Grin2a*$^{+/+}$, *Grin2a*$^{+/-}$, and *Grin2a*$^{-/-}$ juvenile (P14-16) mice. Data represented are mean ± SEM. AP = action potential.

	**AP Probability 1st Stimulation**	**AP Probability 2nd Stimulation**	**AP Probability 3rd Stimulation**	**AP Probability 4th Stimulation**	**AP Probability 5th Stimulation**	**Total Number of APs Generated**
***Grin2a*$^{+/+}$ mice** N=16 cells/5 mice	0.05 ± 0.04	0.17 ± 0.07	0.2 ± 0.07	0.08 ± 0.03	0.05 ± 0.05	2.75 ± 0.91
***Grin2a*$^{+/-}$ mice** N=16 cells/4 mice	0.06 ± 0.02	0.58 ± 0.1	0.29 ± 0.09	0.13 ± 0.05	0.1 ± 0.04	5.88 ± 1.18
***Grin2a*$^{-/-}$ mice** N=16 cells/4 mice	0.03 ± 0.04	0.51 ± 0.1	0.38 ± 0.09	0.21 ± 0.06	0.09 ± 0.04	6.06 ± 1.12

Supplemental Table S5. Passive and action potential firing properties of CA1 pyramidal cells in *Grin2a*$^{+/+}$, *Grin2a*$^{+/-}$, and *Grin2a*$^{-/-}$ juvenile (P14-16) mice. Data represented are mean ± SEM. RMP = resting membrane potential; AP = action potential; depolarization block = current required for depolarization-induced block of AP firing.

	**RMP** (mV)	**Capacitance** (pF)	**Input Resistance** (MΩ)	**Time Constant** (ms)	**Rheobase** (pA)	**Sag Ratio**	**Max AP Firing** (Hz)	**Depolarization Block** (pA)
***Grin2a*$^{+/+}$ mice** N=16 cells/5 mice	-62 ± 1.3	89 ± 5.8	231 ± 22	21 ± 2.4	20 ± 4.4	1.06 ± 0.01	35 ± 2.4	301 ± 35
***Grin2a*$^{+/-}$ mice** N=15 cells/4 mice	-61 ± 1.1	114 ± 12	179 ± 12	21 ± 2.4	23 ± 5.2	1.1 ± 0.01	41 ± 2	389 ± 27
***Grin2a*$^{-/-}$ mice** N=16 cells/4 mice	-63 ± 1.2	106 ± 7.3	190 ± 8.9	20 ± 1.4	19 ± 4	1.1 ± 0.01	40 ± 1.9	391 ± 36

Supplemental Table S6. Parvalbumin-positive CA1 density and cellular lamination in *Grin2a*$^{+/+}$, *Grin2a*$^{+/-}$, and *Grin2a*$^{-/-}$ preadolescent (P21-26) mice. Data represented are mean ± SEM. 4-5 hippocampi from each animal were imaged across 4 animals per genotype for all staining and cell counts.

	**CA1 Cell Density** (# / mm^3)	***stratum oriens*** (% total CA1 PV cells)	***stratum pyramidale*** (% total CA1 PV cells)	***stratum radiatum*** (% total CA1 PV cells)	***stratum lacuosum moleculare*** (% total CA1 PV cells)
***Grin2a*$^{+/+}$ mice** N=17 slices/4 mice	4875 ± 162	42 ± 2.8	51 ± 2.8	7.5 ± 1.5	0
***Grin2a*$^{+/-}$ mice** N=16 slices/4 mice	4542 ± 198	40 ± 2.7	49 ± 2.4	11 ± 8.4	0
***Grin2a*$^{-/-}$ mice** N=20 slices/4 mice	6488 ± 276	40 ± 2.3	52 ± 2.3	9.4 ± 5.4	0

Supplemental Table S7. Cholecystokinin-positive CA1 density and cellular lamination in *Grin2a*$^{+/+}$, *Grin2a*$^{+/-}$, and *Grin2a*$^{-/-}$ preadolescent (P21-26) mice. Data represented are mean ± SEM.

	**CA1 Cell Density** (# / mm^3)	***stratum oriens*** (% total CA1 CCK cells)	***stratum pyramidale*** (% total CA1 CCK cells)	***stratum radiatum*** (% total CA1 CCK cells)	***stratum lacuosum moleculare*** (% total CA1 CCK cells)
***Grin2a*$^{+/+}$ mice** N=20 slices/4 mice	4060 ± 210	24 ± 2.4	11 ± 2.1	47 ± 2.2	17 ± 2.4
***Grin2a*$^{+/-}$ mice** N=16 slices/4 mice	3790 ± 177	23 ± 1.9	10 ± 1.7	50 ± 1.9	17 ± 1.6
***Grin2a*$^{-/-}$ mice** N=20 slices/4 mice	4209 ± 299	27 ± 2.3	16 ± 1.7	45 ± 2.8	12 ± 9

Supplemental Table S8. Electrophysiological properties of wildtype CA1 PV cells during development. Data represented are mean ± SEM. RMP = resting membrane potential; AP = action potential; AHP = afterhyperpolarization of the AP; depolarization block = current required for depolarization-induced block of AP firing.

	**RMP** (mV)	**Capacitance** (pF)	**Input Resistance** (MΩ)	**Time Constant** (ms)	**Rheobase** (pA)	**Sag Ratio**
**Neonate (P6-7)**<br>N=7 cells/3 mice	-57 ± 4.5	193 ± 27	211 ± 28	40 ± 6.4	33 ± 12	1.11 ± 0.02
**Juvenile (P13-15)**<br>N=19 cells/6 mice	-64 ± 1.1	110 ± 6.5	96 ± 6.1	10 ± 0.6	147 ± 12	1.01 ± 0.01
**Preadolescent (P21-26)**<br>N=21 cells/6 mice	-65 ± 1.3	121 ± 7	96 ± 8.3	11 ± 0.9	134 ± 21	0.96 ± 0.05
**Adult (P70+)**<br>N=12 cells/6 mice	-62 ± 1.7	127 ± 13	101 ± 9.7	12 ± 0.8	128 ± 21	0.99 ± 0.01
--	**AP Half-Width** (ms)	**AP Amplitude** (mV)	**AHP Amplitude** (mV)	**Max AP Firing** (Hz)	**Depolarization Block** (pA)	
**Neonate (P6-7)**<br>N=7 cells/3 mice	1.4 ± 0.25	45 ± 4.0	10 ± 2.7	83.9 ± 13	425.7 ± 91	
**Juvenile (P13-15)**<br>N=19 cells/6 mice	0.58 ± 0.02	49 ± 2.0	10 ± 1.1	177 ± 11	1072 ± 87	
**Preadolescent (P21-26)**<br>N=21 cells/6 mice	0.40 ± 0.01	47 ± 1.0	13 ± 0.8	268 ± 13	1355 ± 101	
**Adult (P70+)**<br>N=12 cells/6 mice	0.48 ± 0.02	39 ± 2.5	11 ± 1.0	247 ± 16	1150 ± 161	

Supplemental Table S9. Passive electrophysiological properties during development in *Grin2a*$^{+/+}$, *Grin2a*$^{+/-}$, and *Grin2a*$^{-/-}$ CA1 PV cells. RMP = resting membrane potential.

	**RMP** (mV)	**Capacitance** (pF)	**Input Resistance** (MΩ)	**Time Constant** (ms)	**Sag Ratio**
**Juvenile *Grin2a*$^{+/+}$**<br>N=19 cells/6 mice	-64 ± 1.1	110 ± 6.5	96 ± 6.1	10 ± 0.6	1.01 ± 0.01
**Juvenile *Grin2a*$^{+/-}$**<br>N=19 cells/6 mice	-63 ± 1.6	145 ± 8.1	133 ± 13	19 ± 2.5	1.02 ± 0.01
**Juvenile *Grin2a*$^{-/-}$**<br>N=14 cells/4 mice	-59 ± 3	111 ± 11	184 ± 23	22 ± 4.4	1.04 ± 0.02
**Preadolescent *Grin2a*$^{+/+}$**<br>N=21 cells/6 mice	-65 ± 1.3	121 ± 7	96 ± 8.3	11 ± 0.9	0.96 ± 0.05
**Preadolescent *Grin2a*$^{+/-}$**<br>N=20 cells/5 mice	-61 ± 1.1	126 ± 12	113 ± 9.4	13 ± 1.1	1.01 ± 0.004
**Preadolescent *Grin2a*$^{-/-}$**<br>N=18 cells/5 mice	-63 ± 3.8	117 ± 10	149 ± 17	17 ± 2	1.01 ± 0.05
**Adult *Grin2a*$^{+/+}$**<br>N=12 cells/6 mice	-62 ± 1.7	127 ± 13	101 ± 9.7	12 ± 0.8	0.99 ± 0.01
**Adult *Grin2a*$^{+/-}$**<br>N=12 cells/4 mice	-59 ± 2.5	107 ± 5.8	99 ± 7.9	11 ± 1.0	1.01 ± 0.003
**Adult *Grin2a*$^{-/-}$**<br>N=14 cells/5 mice	-62 ± 1.8	117 ± 9.3	107 ± 12	12 ± 0.8	1.00 ± 0.002

Supplemental Table S10. Action potential waveform and firing properties during development in *Grin2a*$^{+/+}$, *Grin2a*$^{+/-}$, and *Grin2a*$^{-/-}$ CA1 PV cells. AP = action potential; AHP = afterhyperpolarization of the AP; depolarization block = current required for depolarization-induced block of AP firing.

	**Rheobase** (pA)	**AP Half-Width** (mV)	**AP Amplitude** (mV)	**AHP Amplitude** (mV)	**Max AP Firing** (Hz)	**Depolarization Block** (pA)
**Juvenile *Grin2a*$^{+/+}$**<br>N=19 cells/6 mice	147 ± 12	0.58 ± 0.02	49 ± 2.0	10 ± 1.1	177 ± 11	1072 ± 87
**Juvenile *Grin2a*$^{+/-}$**<br>N=19 cells/6 mice	100 ± 14	0.69 ± 0.04	47 ± 1.1	11 ± 1.1	153 ± 12	861 ± 78
**Juvenile *Grin2a*$^{-/-}$**<br>N=14 cells/4 mice	82 ± 22	0.82 ± 0.07	46 ± 4.5	10 ± 1.3	135 ± 15	725 ± 116
**Preadolescent *Grin2a*$^{+/+}$**<br>N=21 cells/6 mice	128 ± 21	0.40 ± 0.01	47 ± 1.0	13 ± 0.8	268 ± 13	1355 ± 101
**Preadolescent *Grin2a*$^{+/-}$**<br>N=20 cells/5 mice	135 ± 21	0.45 ± 0.05	46 ± 2	14 ± 0.6	227 ± 14	1083 ± 108
**Preadolescent *Grin2a*$^{-/-}$**<br>N=18 cells/5 mice	93 ± 19	0.54 ± 0.04	45 ± 3	11 ± 1.6	186 ± 18	761 ± 111
**Adult *Grin2a*$^{+/+}$**<br>N=12 cells/6 mice	128 ± 21	0.48 ± 0.02	39 ± 2.5	11 ± 1.0	247 ± 16	1150 ± 161
**Adult *Grin2a*$^{+/-}$**<br>N=12 cells/4 mice	103 ± 13	0.46 ± 0.03	46 ± 2.1	12 ± 0.6	225 ± 20	1145 ± 124
**Adult *Grin2a*$^{-/-}$**<br>N=14 cells/5 mice	130 ± 19	0.45 ± 0.02	38 ± 3.3	10 ± 0.9	244 ± 18	1161 ± 170
