# Supplementary material for: Loss of Grin2a causes a transient delay in the electrophysiological maturation of hippocampal parvalbumin interneurons
Source: Commun Biol. 2023 Sep 19;6:952. doi: 10.1038/s42003-023-05298-9 (PMC10507040; doi:10.1038/s42003-023-05298-9)
Supplement: Supplementary file 2 — Description of Additional Supplementary Files [file 42003_2023_5298_MOESM2_ESM.pdf]

Description of Additional Supplemental Files

File Name: Supplemental Data 1
Description: Source Data for Figures 1 and 2.
Human GRIN2A patient data along with data for evoked NMDA receptor EPSCs and evoked action-potential spiking data from CA1 pyramidal cells from juvenile mice.

File Name: Supplemental Data 2
Description: Source Data for Figures 3 and 4.
CA1 cell density counts and percent cells per CA1 layer for both anti-parvalbumin and anti-cholecystokinin immunohistochemical experiments from preadolescent mice.

File Name: Supplemental Data 3
Description: Source Data for Figures 5-8.
Electrophysiological data from CA1 PV cells from neonatal, juvenile, preadolescent, and adult mice
